# Association between Periodontitis and HbA1c Levels in Non-Diabetic Patients: A Systematic Review and Meta-Analysis

**DOI:** 10.3390/healthcare11192649

**Published:** 2023-09-28

**Authors:** Dan Zhao, Yangyang Sun, Xin Li, Xiaoxiao Wang, Lijie Lu, Chen Li, Yaping Pan, Songlin Wang

**Affiliations:** 1 Department of Implant Dentistry, Beijing Stomatological Hospital, Capital Medical University, Beijing 100050, China; zhaodanashley@hotmail.com; 2 Department of Periodontics, School of Stomatology, China Medical University, Shenyang 110002, China; sunyangyang_cmu@163.com (Y.S.); ljlu1245672856@163.com (L.L.); lichen@cmu.edu.cn (C.L.); yppan@cmu.edu.cn (Y.P.); 3 School of Public Health, National Institute for Data Science in Health and Medicine, Capital Medical University, Beijing 100069, China; elaine@mail.ccmu.edu.cn; 4 Department of Oral Implantology and Prosthodontics, Shenzhen Stomatology Hospital Affiliated to Shenzhen University, Shenzhen 518001, China; wxiaox7@163.com; 5 Beijing Key Laboratory of Tooth Regeneration and Function Reconstruction, Beijing Laboratory of Oral Health and Beijing Stomatological Hospital, Capital Medical University, Beijing 100050, China

**Keywords:** HbA1c, early diagnosis, periodontitis, hyperglycemia, diabetes mellitus, systematic review, meta-analysis

## Abstract

Background: A high detection rate of diabetes among dental visitors has been reported recently. This systematic review aimed to evaluate the association between periodontitis and glycated hemoglobin (HbA1c) levels among non-diabetic individuals. Methods: The EMBASE, MEDLINE, Web of Science, Cochrane Library, PubMed, and Open GREY databases were searched, and observational studies published until 1st June 2023 were identified. A methodological quality assessment was conducted based on the original and modified versions of the Newcastle–Ottawa scale. Cohort, case–control, and cross-sectional studies that performed clinical periodontal examinations and measured HbA1c levels in non-diabetic adults were included. A meta-analysis was conducted to estimate the weighted mean difference (WMD) between individuals with and without periodontitis. Results: In total, 29 case–control and 5 cross-sectional studies were selected from 2583 potentially eligible articles. Among them, sixteen case–control and three cross-sectional studies with moderate to high quality were selected for the meta-analyses. The HbA1c levels in periodontitis patients were significantly higher than those in individuals with healthy periodontal conditions (WMD = 0.16; *p* < 0.001) among the non-diabetic populations. Conclusions: This study reveals a significant association between periodontitis and HbA1c levels in non-diabetic populations. Thus, HbA1c screening may be recommended to detect potential hyperglycemia in non-diabetic periodontitis patients.

## 1. Introduction

Periodontitis is one of the most common inflammatory diseases, characterized by the dysbiosis of periodontal bacteria and host immune response [1]. Additionally, it is the most common cause of natural tooth loss in adults [2]. The prevalence of periodontitis is nearly 50% among adults worldwide, and this proportion is even higher (70%) among those over 65 years of age, which is a cause of concern in terms of global health and the economic burden [3,4,5]. Furthermore, periodontitis can elevate systemic inflammatory pressure and impact systemic health [6,7,8,9].

Diabetes mellitus (DM) is a common chronic metabolic disease characterized by sustained hyperglycemia; it is widely accepted as a major complication of periodontitis [6,10]. The underlying mechanisms between periodontitis and increased glycated hemoglobin (HbA1c) levels have been investigated for several decades. The evidence indicates that chronic systemic inflammation contributes to insulin resistance, which leads to increased HbA1c levels and, subsequently, diabetes and its complications [10,11]. The levels of several inflammatory mediators are significantly increased in the circulation of periodontitis patients and decreased after effective periodontal debridement in periodontitis patients [12,13]. For instance, it has been shown that tumor necrosis factor-α can interrupt insulin signaling mechanisms and reduce the entrance of glucose into cells [14]. Therefore, the long-standing systemic inflammation in periodontitis patients may be a biological plausibility that promotes poor glycemic control and contributes to the progression of DM. Furthermore, periodontitis is known to have a negative impact on the outcomes of DM [15,16]. Severe periodontitis can contribute to poor glycemic control and compromise the response to diabetes management [17].

The bidirectional relationship between periodontitis and DM has been demonstrated in the past [18]. Diabetic patients have an approximately three-fold higher risk of developing periodontitis than non-diabetics [19]. Patients with poor glycemic control are more likely to have a higher prevalence of severe periodontitis, which suggests that diabetes management may be a significant predictor of periodontitis development [19,20].

The biomarker HbA1c is used to assess the formation of sugar-coated hemoglobin and determine the presence of excessive glucose in the bloodstream. It has been utilized as a diagnostic tool to evaluate the average glycemic level. Evidence suggests that effective periodontal treatment can lower the HbA1c and circulating C-reactive protein levels [21,22,23,24].

Previous studies mainly focused on the association between periodontitis and the glycemic level in patients with DM [25,26]; however, evidence of this association among those without DM is scarce. Impaired immune response and enhanced systemic inflammation are commonly observed in patients with periodontitis and/or DM [27]. Thus, it is vital to investigate the role of periodontitis in the onset and development of DM, especially in the non-diabetic population. The aim of this study was to identify observational studies that performed clinical periodontal examinations and measured the HbA1c levels in non-diabetic adults. A meta-analysis was performed to estimate the weighted mean difference (WMD) in HbA1c levels between individuals with and without periodontitis. The following PECO question was proposed: among individuals without diabetes, are HbA1c levels different between those with and without periodontitis?

## 2. Materials and Methods

The systematic review and meta-analysis were conducted based on the Preferred Reporting Items for Systematic Reviews and Meta-Analyses (PRISMA) guidelines [28]. The proposal for the study was registered at the National Institute for Health Research, International Prospective Register of Systematic Reviews (PROSPERO; registration number, CRD42021249010).

Electronic searches of studies published until 1 June 2023 were conducted in five publication databases, including EMBASE, MEDLINE, Web of Science, Cochrane Library, and PubMed. There were no restrictions on the publication date and language, and unpublished articles were identified in Open GREY. One reviewer (SYY) performed the searches based on the search strategies developed by another reviewer (DZ) with the assistance of a university research librarian. The search criteria for all databases are presented in Table S1. References in the included publications were hand-searched to identify additional potential studies. 

The two reviewers (DZ and SYY) independently screened the titles and abstracts of all the related publications and grouped the potentially eligible articles for further evaluation. The included papers were then carefully verified in full text, and reviewer disagreements were resolved through discussion. If an agreement could not be reached, a third reviewer (SLW) decided whether to include or exclude the article in this review. 

The eligibility criteria for this systematic review were as follows:

Patients (P): adults without DM, which was confirmed by self-reporting, medical history, or glycemic tests. Studies comprising non-DM individuals recruited to match the DM patients were not accepted.

Exposure (E): periodontitis diagnosed based on at least one clinical parameter; self-reporting was unacceptable.

Control (C): individuals without periodontitis.

Study outcomes (O): HbA1c measurements; other glycemic test results were considered as secondary outcomes.

Study design (S): (i) observational studies or baseline data of interventional studies; (ii) those with no less than 10 subjects with periodontitis; and (iii) all publications, including in-press/online and unpublished studies. 

Data extraction was performed independently by the two reviewers (DZ and SYY). It included the study characteristics (first author, publication year, and study design), population characteristics (sample size, demographic data, and description of subjects without DM), the definition of periodontitis, and outcomes (HbA1c levels and other parameters assessing glycemic status). Details about the origin of the subjects, periodontal examinations, covariates, and clinical assessment methods (blinding, training, and calibration) were also collected (Table S2).

The original and modified versions of the Newcastle–Ottawa Scale (NOS) were used to assess the quality of the cohort/case–control and cross-sectional studies (Table S3), respectively [29,30,31]. A star was allotted if the study met the criteria for high quality, and the methodological quality of each study was rated based on the proportion of stars obtained. Only moderate- to high-quality papers with scores of 51% or higher were included in the meta-analysis [32]. 

Stata version 16.0 was used to conduct the meta-analyses based on the study design. Estimates of the pooled WMD were analyzed using the random-effects inverse-variance model. A forest plot was created to summarize the estimated effects and 95% confidence interval (CI) of all the included articles. The statistical heterogeneity was explored using the Chi-square-based Cochrane’s Q statistic (*p* < 0.05) and I^2^ (>50%). Egger’s [33] and Begg’s [34] tests and the funnel plots were used to demonstrate publication bias. Influence analysis was employed using the one-by-one elimination method to estimate the stability of the results. Meta-regression and subgroup analyses were performed to investigate the potential heterogeneity using study design, gender, body mass index (BMI), smoking status, and the severity of periodontitis as the independent variables.

## 3. Results

### 3.1. Search Results

**Figure 1:**
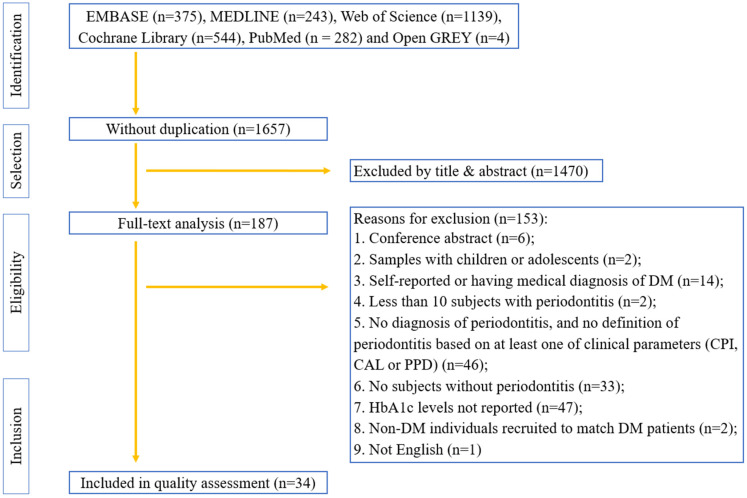
Flowchart of the study selection.

A total of 2583 related publications and 4 unpublished studies were identified from the six electronic databases. After removing 930 duplications, the titles and abstracts of the 1470 remaining articles were screened, and 187 were selected for full-text analysis. Finally, 34 studies that met the predetermined eligibility criteria were included in the systematic review (Figure 1).

### 3.2. Study Characteristics

Table 1 summarizes the characteristics of the 5 cross-sectional studies [35,36,37,38,39] and 29 case–control studies published between 2008 and 2023. These studies evaluated the association between periodontitis and HbA1c levels among subjects without diabetes. Most of the data were derived from convenience samples in hospitals. Two studies contained data from the Korean National Health and Nutrition Examination Survey conducted in 2012 and 2013–2015 [37,38], and one recruited the community sample [40]. The number of participants in the included studies varied from 28 to 8341; two case–control studies provided details on the power calculation [41,42]. The average age of the included subjects ranged from 29.8 to 56.5 years. One study [40] recruited only males, and six studies [43,44,45,46,47] did not provide information about the gender of the participants; the percentage of males ranged from 20% to 87.1% in the remaining twenty-seven studies. Two studies recruited only those with normal BMI [48,49], whereas five studies comprised non-obese patients only [46,50,51,52,53]. Furthermore, 73.5% (25/34) of the studies excluded smokers or tobacco users. More than half (19/34) of the studies defined non-DM subjects as systematically healthy individuals, and one study categorized participants free of severe systemic diseases as the non-DM group [54]. The remaining studies (14/34) used the HbA1c level, fasting plasma glucose (FPG) level, fasting blood sugar level, random blood sugar (RBS) level, and/or oral glucose tolerance test (OGTT) to describe the non-DM group; multiple glycemic testes were simultaneously applied in 8 of these 14 studies [35,43,48,50,53,55,56,57].

The definitions of periodontitis differed among the studies included in this review (Table 1). The American Academy of Periodontology (AAP) 1999 classification was used to diagnose periodontitis in 20 (58.8%) studies, whereas 4 studies [35,58,59,61] used the American Academy of Periodontology and European Federation of Periodontology (AAP/EFP) 2017 classification; meanwhile, 2 studies [37,38] used the Community Periodontal Index (CPI), and in 1 study [50], the disease was diagnosed based on the criteria from the Center for Disease Control and Prevention and the American Academy of Periodontology (CDC/AAP). Six sites per tooth were examined in more than half of the studies, whereas four sites per tooth were examined in two studies [47,66]. Twelve studies did not describe the details of the specific sites examined. Full-mouth periodontal examinations were performed in more than half of the included studies (19/34), and the third molars 32.4% were skipped in 11 (32.4%) studies. A single examiner conducted the examinations in 55.9% (19/34) of the studies, and the training or calibration method was used in half of these studies. Four studies [36,53,55,66] employed two examiners and reported that the examiners were well-trained or calibrated. The examiners were blinded in four studies [40,48,59,60]. The criteria for the minimum number of remaining teeth were mentioned in 82.4% (28/34) of the studies (range, 10–20). Patients who had undergone periodontal therapy within the past 90 days to 1.5 years were excluded in 76.5% (26/34) of the studies (Table S2).

Almost all the studies, except for three [35,39,59], compared the HbA1c levels between the periodontitis and control groups (Table 1). Among them, 10 reported that the HbA1c level in the periodontitis group was higher than that in the control group (*p* < 0.05) [37,38,42,43,44,45,46,50,54,57], and only 2 of them employed the normality test before the comparative analysis (Table S2) [46,57]. Five studies considered the confounding effects of covariates (including age, gender, BMI, and smoking habits) [37,41,47,51,66], and multivariate analysis was conducted in two of these studies [37,41]. 

### 3.3. Methodological Quality

The quality of each study was assessed in accordance with the original and modified versions of the NOS (Table S4). Two out of five cross-sectional studies and none of the case–control studies were rated as high-quality [37,38]; one cross-sectional [36] and sixteen case–control studies [40,41,42,47,48,49,50,51,53,54,56,57,59,62,66,68] were rated as moderate-quality, with the main source of bias coming from the selection of controls. 

### 3.4. Meta-Analysis

**Figure 2:**
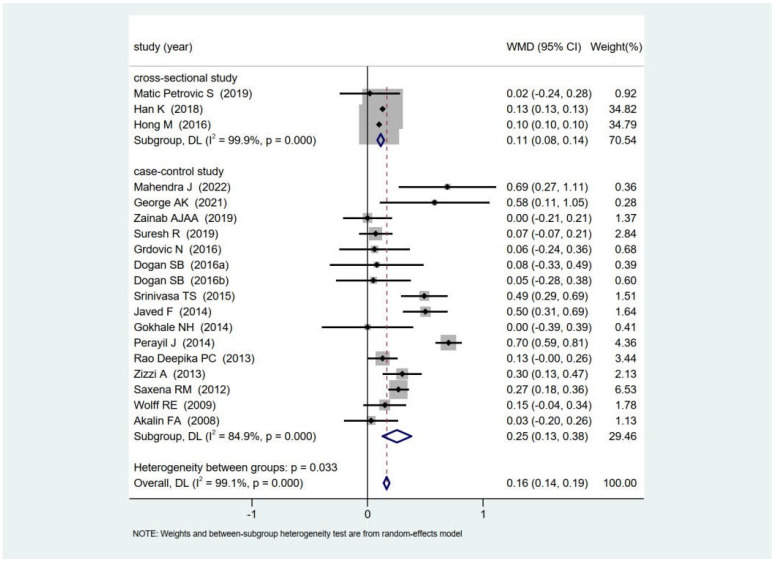
Comparison of the HbA1c levels between non-DM subjects with and without periodontitis according to different study designs [36,37,38,40,41,42,47,48,49,50,51,53,54,56,57,59,62,66,68]. WMD: weighted mean difference; DL: DerSimonian–Laird.

**Figure 3:**
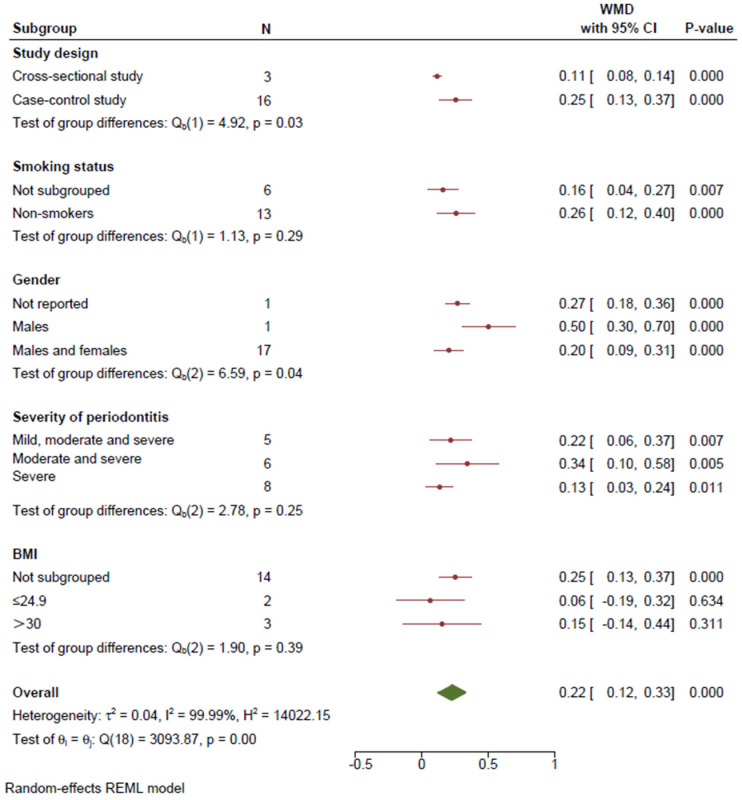
Subgroup analysis of HbA1c levels between non-DM subjects with and without periodontitis based on study design, smoking status, gender, severity of periodontitis, and BMI. BMI: body mass index; WMD: weighted mean difference.

Three cross-sectional studies [36,37,38] and sixteen case–control studies [40,41,42,47,48,49,50,51,53,54,56,57,59,62,66,68] were selected for the meta-analysis (random-effects model) based on the methodological quality. Although the heterogeneity was high (*I*^2^ = 99.1%; *p* < 0.001), periodontitis was associated with HbA1c levels in non-diabetic subjects in the observational studies (WMD = 0.16; 95% CI = 0.14–0.19; *p* < 0.001; Figure 2). Meta-regression was conducted to identify the sources of heterogeneity, and the results yielded a model with no statistically significant difference (*F* = 1.37; *p* = 0.306). As shown in Figure 3, subgroup analysis showed statistically significant intergroup differences in the study design (*Q* = 4.92; *p* = 0.03) and gender (*Q* = 6.59; *p* = 0.04). Despite the significant heterogeneity, the HbA1c levels in periodontitis patients were significantly higher than those in individuals with healthy periodontal conditions in the cross-sectional (WMD = 0.11; 95% CI = 0.08–0.14; *p* < 0.001) and case–control (WMD = 0.25; 95% CI = 0.13–0.38; *p* < 0.001) studies (Figure 2). 

Two symmetrical funnel plots were generated from the meta-analyses, suggesting no publication bias (cross-sectional studies, Figure S1; case–control studies, Figure S2). The results of the Begg’s and Egger’s tests supported the absence of any significant publication bias in the cross-sectional and case–control studies. The influence analysis indicated that the pooled estimates would not be notably affected in the cross-sectional (Figure S3) and case–control (Figure S4) studies.

## 4. Discussion

This systematic review is the first to summarize the evidence on the association between periodontitis and HbA1c levels in the non-diabetic population based on observational studies. All the studies included in this review were cross-sectional and case–control, suggesting low to moderate levels of evidence in terms of the hierarchy of the sources of evidence [69,70]. The majority of the studies compared the HbA1c levels between individuals with and without periodontitis, but most failed to demonstrate significant findings. However, the meta-analysis in the current review shows that despite the high heterogeneity, the HbA1c levels in non-diabetic patients with periodontitis were significantly greater than those in individuals with healthy periodontal conditions. Subgroup analysis suggested that study design and gender as the potential sources of heterogeneity. Thus, two additional meta-analyses were conducted separately. Interestingly, the significant differences in HbA1c levels were observed between those with and without periodontitis remained in the cross-sectional and case–control studies.

Statistical analyses comparing differences between individuals with or without periodontitis were not conducted in two [35,39] out of the five cross-sectional studies. Of the remaining three studies, two from Korea [37,38] found a significant association between periodontitis and HbA1c levels; the data were obtained from the Korean National Health and Nutrition Examination Survey. One of these Korean studies conducted multivariate logistic regression to adjust the confounders, but no independent association was detected [37]. Although the remaining single study from Serbia [36] failed to report a significant relationship between periodontitis and HbA1c levels, it showed an association between periodontitis and FPG, which may result from the variability in FPG relative to HbA1c [71]. Diagnostic screening can be performed using FPG or HbA1c levels [72]. HbA1c reveals the average blood glucose level over the previous three months because it remains stable on a daily basis [73]. Therefore, it is not always completely correlated with the average blood glucose level [74]. Moreover, the HbA1c level is less impacted by diet when compared to other glycemic biomarkers. Thus, this assay can be conducted without fasting.

Statistical analysis was performed in 96.4% (27/28) of the case–control studies, and 33.3% (9/27) reported statistically significant associations between periodontitis and HbA1c levels. One revealed that covariates, such as age, gender, BMI, and smoking habit, could mask the significant correlation between periodontitis and HbA1c [41]. These risk factors, which are common for both periodontitis and DM, could impact the research findings. Notably, the covariates were considered in almost all the studies included in the current review; however, they were addressed in only 13.8% (4/29) of the studies. One study employed the multivariate linear regression model [41], one matched the gender during the recruitment of participants [51], and the other two studies divided the subjects based on the BMI and analyzed the association between periodontitis and HbA1c levels in the subgroups [47,66]. More than half of the studies (58.6%; 17/29) did not employ normality tests before performing the parametric tests. Incorrect selection of the statistical method during statistical analyses can impact the internal validity of the results. Furthermore, blinding, training, and calibrating the investigators can reduce information bias [75]. However, the investigators were blinded in only four studies [40,48,59,60], and training and calibration were performed in five studies [40,49,60,61,64]. Two trained and calibrated investigators were employed in three studies, which may have reduced the measurement bias during the operational process [53,55,66].

The description of non-diabetes varied among the studies included in this review. More than half of the studies (19/34) defined non-diabetics subjects as systematically healthy individuals or those without a diagnosis of DM [36,37,39,40,41,45,46,47,51,52,58,59,60,63,64,65,66,67,68]. Furthermore, the glycemic test parameters and cut-off values varied from 6% to 6.5% for HbA1c [35,43,48,49,50,53,55,56,57,61,76], 100 to 126 mg/dl for FPG [35,38,42,44,48,50], and 140 to 200 mg/mL for RBS [43,56,62]. These discrepancies can affect the actual glycemic conditions of the participants and increase the heterogeneity among studies, which can further impact the validity of the findings. Similarly, the definition of periodontitis was inconsistent among the studies. Four common classifications (i.e., CPI, AAP 1999, CDC/AAP, and AAP/EFP 2017) were used in 27 out of 34 studies; the remaining 7 diagnosed periodontitis using their own definitions [36,39,44,52,56,63,65]. Furthermore, conflicting findings were observed among studies despite using the same criteria. Many studies used modified versions of the AAP 1999 case definition by adding parameters such as plaque index, gingival index, modified sulcus bleeding index, and radiographic bone loss [40,43,45,48,49,51,57,60,62,67]. The different diagnostic criteria used could influence the severity of periodontitis, increase heterogeneity, and affect the results of the current study. Almost half of the studies recruited patients with severe periodontitis only, which may lead to an overestimation of the association between periodontitis and HbA1c levels. Moreover, examining four sites per tooth could result in underestimating the severity of periodontitis [47,66].

The origin of the sample can impact the external validity of this systematic review. The two cross-sectional studies with significant association were conducted in Korea [37,38], and almost all the case–control studies with significant association (7/8) were conducted in India [42,43,44,45,46,50,54]. The majority of the studies used convenience samples from hospitals, except for three, which recruited community samples [37,38,40]. Therefore, the findings of the review should be interpreted with caution.

This review has some limitations. Firstly, only cross-sectional and case–control studies were included. The lack of a prospective study can weaken the strength of evidence in this study. Thus, the inclusion of cohort studies to detect a potential directional relationship is warranted in the future. Secondly, 44.1% of the studies included in this review were of low quality. The exclusion of these articles from the quantitative analysis decreased the sample size and the diversity of the demographic characteristics, which may have impacted the external validity of the results of the current meta-analysis. Thirdly, the high heterogeneity caused by variations in the definitions of non-diabetes and periodontitis may have impacted the real glycemic and periodontal condition of subjects and, consequently, influenced the comparability and confidence in the pooled estimates. The recruitment of subjects with various periodontal statuses is required to validate the findings of this review and provide evidence on the potential dose effect of periodontitis on HbA1c levels. Finally, the majority of studies recruited convenience samples. Thus, additional community samples, including subjects from various races, are expected to make the findings more representative of the broader population.

## 5. Conclusions

The results from the meta-analyses identified significantly higher levels of HbA1c among non-diabetic periodontitis patients compared to those in non-diabetic individuals with healthy periodontal conditions. Periodontitis and DM can act as comorbidities; therefore, prospective studies using representative community samples should be conducted to improve the level of evidence and clarify whether the association is directional or non-directional. Nonetheless, the present meta-analysis shows that a healthy periodontal status has a beneficial effect on the glycemic condition in healthy individuals. The findings of this study may help general dental practitioners and periodontists to focus on general health and encourage them to collaborate closely with medical professionals. The emerging co-management care scheme will help improve the oral health and the general well-being of patients who visit the dental clinic.

## Figures and Tables

**Table 1:** Data collection of cross-sectional and case–control studies.

First Author (Publication Year)	Study Design *	Sample Size	Demographic Data of Subjects	Description of Subjects without DM	Definition of Periodontitis	Glycemic State
Gomathi GD (2023) [58]	Case–control study, convenience sample	Stage II periodontitis group and periodontal health group: 15 vs. 15	Non-tobacco chewers and non-smokers	The ADA criteria.	AAP/EFP 2017 classification.	In periodontitis group and periodontal health group, HbA1c (%, mean ± SD): 5.54 ± 0.29 vs. 5.3 ± 0.26, *p* > 0.05; FPG (mg/dL, mean ± SD): 92.3 ± 5.76 vs. 92.0 ± 4.85, *p* > 0.05; by one-way ANOVA.
Mahendra J (2022) [59]	Case–control study, convenience sample	Periodontitis group and control group: 30 vs. 30	Patients with periodontitis mean aged 54.03 ± 9.10 years with 6/30 (20.6%) males, healthy controls mean aged 37.63 ± 10.26 years with 7/30 (22.2%) males	With systemic health and without systemic conditions.	AAP/EFP 2017 classification. Periodontitis: interdental CAL was detectable at >2 nonadjacent teeth with CAL ≥ 3 mm and PD > 3 mm present in >2 teeth.	In periodontitis group and control group, HbA1c (%, mean ± SD): 5.97 ± 1.16 vs. 5.28 ± 0.22; FBS (mg/dL, mean ± SD): 111.17 ± 48.08 vs. 89.43 ± 9.72; by ANOVA. The difference of periodontitis group with control group was not compared.
George AK (2021) [50]	Case–control study, convenience sample	Severe periodontitis group and control group: 37 vs. 37	Patients with periodontitis mean aged 43.35 ± 7.70 years with 20/37 (54.1%) males, healthy controls mean aged 40.70 ± 6.19 years with 13/37 (35.1%) males. Non-smokers; non-obese (BMI < 30 kg/m^2^)	FBS < 126 mg/dL, HbA1c < 6.5%.	CDC/AAP 2013 case definition. Severe periodontitis: ≥2 interproximal sites with CAL ≥ 6 mm (not on the same tooth) and ≥1 interproximal site with PD ≥ 5 mm.	In periodontitis group and control group, HbA1c (%, mean ± SD): 5.87 ± 1.46 vs. 5.29 ± 0.20, *p* = 0.021; FBS (mg/dL, mean ± SD): 96.81 ± 23.59 vs. 83.27 ± 8.28, *p* = 0.002; by unpaired *t*-test.
Akram Z (2020) [60]	Case–control study, convenience sample	Periodontitis group and periodontal health group: 31 vs. 31	Patients with periodontitis mean aged 51.5 (42–54) years with 25/31 (80.6%) males, healthy controls mean aged 50.7 (46–58) years with 27/31 (87.1%) males. Non-smokers (including electronic cigarettes)	Self-reported systemically healthy individuals.	AAP 1999. Periodontitis: PI, BOP, PD ≥ 4 mm, CAL ≥ 3 mm and marginal BL ≥ 3 mm in at least 30% of sites. Periodontal health group: subjects without chronic periodontitis.	In periodontitis group and periodontal health group, mean (range) of HbA1c (%): 4.7 (4.2–5.1) vs. 4.2 (4.1–4.5), *p* > 0.05; FPG (mg/dL, mean ± SD): 97.2 ± 11.7 vs. 94.9 ± 6.7, *p* > 0.05; by one-way ANOVA and Bonferroni post hoc adjustment tests.
Altingoz SM (2020) [61]	Case–control study, convenience sample	Stage III periodontitis group and periodontal health group: 26 vs. 28	Patients with periodontitis mean aged 46.1 ± 5.3 years with 8/26 (30.8%) males, healthy controls mean aged 44.8 ± 11.5 years (in the text) and 44.8 ± 6.5 years (in Table 1) with 10/28 (35.7%) males	No definition; for reference, Type 2 DM: diagnosed by physicians for at least the past 5 years and 6.5% ≤ HbA1c < 12%.	AAP/EFP 2017 classification. Periodontitis: ≥ 8 sites with PD ≥ 6 mm, ≥4 sites with CAL ≥ 5 mm, distributed in at least 2 different quadrants. Periodontal health group: negative periodontal disease history, PD ≤ 3 mm and CAL ≤ 1 mm, without clinical signs of gingival inflammation and with good oral hygiene.	In periodontitis group and periodontal health group, HbA1c (%, mean ± SD): 4.8 ± 0.3 vs. 4.7 ± 0.2, *p* > 0.05; FPG (mg/dL, mean ± SD): 97.2 ± 11.7 vs. 94.9 ± 6.7, *p* > 0.05, by the ANOVA test with a Bonferroni correction.
Yilmaz D (2020) [35]	Cross-sectional study, convenience sample	Periodontitis group and periodontal health group: 29 vs. 28	Patients with periodontitis mean aged 45.4 ± 12.9 years and 84.7% were males, healthy controls mean aged 43.9 ± 14.4 years and 82.7% of 28 healthy subjects were males. Non-smokers	Metabolic health: FPG <126 mg/dL (7.0 mmol/L, fasting is defined as no caloric intake for at least 8 h) and HbA1c <6.5% (48 mmol/mol) based on ADA 2018 guideline.	AAP/EFP 2017 classification. Periodontitis: BOP ≥ 10% and interdental CAL was detectable at ≥2 non-adjacent teeth with PD ≥ 4 mm. Periodontal health: BOP ˂ 10% of the surfaces and no sites with PD > 3 mm besides no CAL or BL.	In periodontitis group and periodontal health group, HbA1c (%, mean ± SD): 5.41 ± 0.54 vs. 5.56 ± 0.45; FPG (mg/dL, mean ± SD): 97.8 ± 12.1 vs. 95.6 ± 10.1. The difference between two groups was not compared.
Agrawal AA (2019) [43]	Case–control study, convenience sample	Chronic periodontitis group, chronic gingivitis group, and periodontal health group: 20	Patients with periodontitis mean aged 39.29 years, with gingivitis mean aged 41.45 years, with healthy periodontal status mean aged 44.65 years. Non-tobacco chewers and non-smokers	HbA1c levels < 6.5%, RBS levels ≤ 200 mg/dL.	AAP 1999. Periodontitis: GI ≥ 1, PI ≥ 1, PD ≥ 5 mm and CAL ≥ 5 mm. Gingivitis: GI ≥ 1, PI ≥ 1, PD ≤ 3 mm. Periodontal health: GI < 1, PI < 1, PD ≤ 3 mm.	HbA1c (%, mean ± SD) in periodontitis group was 6.60 ± 1.66; in gingivitis group was 4.67 ± 0.37; in periodontal health group was 4.63 ± 0.36, *p* < 0.05. RBS (mg/dL, mean ± SD) in periodontitis group was 169.76 ± 41.59; in gingivitis group was 180.35 ± 22.27; in periodontal health group was 97.65 ± 47.47, *p* > 0.05; by Student’s unpaired *t*-test.
Matic Petrovic S (2019) [36]	Cross-sectional study, convenience sample	Chronic periodontitis group and periodontal health group: 42 vs. 36	Patients with periodontitis mean aged 48 ± 11 years with 17/42 (40.5%) males, healthy controls mean aged 43 ± 4 years with 15/36 (41.7%) males	Free of systemic diseases.	Periodontitis: CAL ≥ 1 mm and PD > 3 mm at > 30% of sites. periodontal health group: PD < 3 mm, CAL = 0 mm, BOP < 25%.	In periodontitis group and periodontal health group, HbA1c (%, mean ± SD): 4.82 ± 0.561 vs. 4.80 ± 0.607, *p* > 0.05; FPG (mmol/L, mean ± SD): 4.97 ± 0.578 vs. 4.64 ± 0.534, *p* < 0.05; by Mann–Whitney U test.
Suresh R (2019) [62]	Case–control study, convenience sample	Chronic moderate periodontitis group and periodontal health group: 20 vs. 20	Patients with periodontitis mean aged 44 ± 7.20 years with 16/20 (80.0%) males, healthy controls mean aged 48 ± 7.13 years with 10/20 (50.0%) males. Non-smokers	Without non-insulin dependent DM, RBS levels < 140 mg/dL.	AAP 1999. Periodontitis: CAL 3–4 mm, OHI-S score 1.3 to 3, GI score 1.1 to 2, PD ≥ 3 to ≤5 mm, CAL ≥ 3 to ≤4 mm in 5 or more teeth. Periodontal health group: OHI-S score 0.8 to 1, GI score 0.1 to 1, PD ≤ 3 mm and with no CAL.	In periodontitis group and periodontal health group, HbA1c (%, mean ± SD): 5.74 ± 0.19 vs. 5.67 ± 0.27, *p* > 0.05, by ANOVA.
Zainab AJAA (2019) [51]	Case–control study, convenience sample, with equal number of males and females	Chronic periodontitis group and periodontal health group: 20 vs. 20	Patients with periodontitis mean aged 43.5 ± 4.81 years with 10/20 (50.0%) males, healthy controls mean aged 40.05 ± 5.56 years with 10/20 (50.0%) males. Never-smokers; non-obese	Without DM or any other systemic disease.	AAP 1999. Periodontitis: ≥5 mm of CAL present at more than 30% of the sites and BL radiographically. Periodontal health group: PD ≤ 3 mm, no CAL, absence of BOP, no radiographic BL.	In periodontitis group and periodontal health group, HbA1c (%, mean ± SD): 4.85 ± 0.39 vs. 4.85 ± 0.29, *p* > 0.05; FPG (mg/dL, mean ± SD): 107.00 ± 9.28 vs. 106.35 ± 8.22, *p* > 0.05, by ANOVA.
Acharya AB (2018) [52]	Case–control study, convenience sample	Chronic periodontitis group and periodontal health group: 20 vs. 20	Patients with periodontitis mean aged 42.09 ± 6.45 years with 12/20 (60.0%) males, healthy controls mean aged 42.80 ± 4.81 years with 10/20 (50.0%) males. Non-tobacco users; BMI < 30 kg/m^2^ and lipid profile in normal limits	Not previously diagnosed with DM.	Periodontitis: generalized BOP, PD of ≥ 5 mm, CAL ≥ 2 mm supplemented by radiographic confirmation of alveolar BL.	In periodontitis group and periodontal health group, HbA1c (%, mean ± SD): 5.72 ± 0.33 vs. 4.87 ± 0.42, *p* > 0.05; RBS (mg/dL, mean ± SD): 116.80 ± 15.98 vs. 105.85 ± 11.398, *p* > 0.05; by Wilcoxon sign–rank tests.
Han K (2018) [37]	Cross-sectional study, nationally representative community sample from multi-centers	Periodontitis group and periodontal health group: 1968 vs. 6373	Patients with periodontitis mean aged 49.29 ± 0.37 years and 56.24% were males, healthy controls mean aged 37.94 ± 0.23 years and 44.65% were males	Without diagnosis of DM.	Periodontitis: CPI ≥ 3.	In periodontitis group and periodontal health group, HbA1c (%, mean ± SD): 5.59 ± 0.01 vs. 5.46 ± 0.01, *p* < 0.05; FPG (mg/dL, mean ± SD): 94.88 ± 0.24 vs. 91.66 ± 0.15, *p* < 0.05; by independent *t*-test. Multivariate logistic regression: adjusted age, gender, smoking, drinking, education, income, and BMI, HbA1c was not significantly different in periodontitis group and periodontal health group.
Dogan SB (2016a) [49]	Case–control study, convenience sample	Chronic periodontitis group and periodontal health group: 15 vs. 15	Patients with periodontitis median aged 48.00 years with 8/15 (53.3%) males, healthy controls median aged 52.00 years with 7/15 (46.7%) males. Never-smokers; BMI ≤ 24.9 kg/m^2^	HbA1c levels < 6.5%.	AAP 1999. Periodontitis: Radiographic signs of BL and CAL, at least 6 teeth with a PD ≥ 5 mm. These teeth showed BOP across a minimum of 2 separate quadrants, and had GI > 1. Periodontal health group: PD ≤ 3 mm, GI = 0, and no indication of CAL or radiographic evidence of alveolar BL (i.e., the gap between the CEJ and bone crest was <3 mm at > 95% of the proximal tooth sites).	In periodontitis group and periodontal health group, HbA1c (%, median): 5.30 vs. 5.10, *p* > 0.05; by Kruskal–Wallis nonparametric test.
Dogan SB (2016b) [48]	Case–control study, convenience sample	Chronic periodontitis group and periodontal health group: 20 vs. 20	Patients with periodontitis median aged 49.50 years with 10/20 (50.0%) males, healthy controls mean aged 50.50 years with 11/20 (55.0%) males. Never-smokers; BMI ≤ 24.9 kg/m^2^	HbA1c <6% and FPG < 100 mg/dL.	AAP 1999. Periodontitis: At least 6 teeth with CAL and a PD ≥ 5 mm. positive BOP within multiple regions. BL affected >30% of existing teeth on clinical and radiographic examination. GI ≥ 2. Periodontal health group: PD ≤ 3 mm, GI = 0, and no indication of CAL or radiographic evidence of alveolar BL (i.e., the gap between the CEJ and bone crest was <3 mm at >95% of the proximal tooth sites).	In periodontitis group and periodontal health group, HbA1c (%, median): 5.10 vs.5.00, *p* > 0.05; FPG (mg/dL, median): 87.00 vs. 87.50, *p* > 0.05; by Kruskal–Wallis non-parametric test.
Grdovic N (2016) [53]	Case–control study, convenience sample	Chronic periodontitis group and periodontal health group: 29 vs. 21	Patients with periodontitis mean aged 48.17 ± 13.48 years with 16/29 (55.2%) males, healthy controls mean aged 33.43 ± 5.28 years with 8/21 (38.1%) males. BMI 19–30 kg/m^2^	Normal parameters on OGTT and HbA1c < 6.5%.	AAP 1999. Periodontitis: CAL > 1 mm and PD > 3 mm at least at three sites in two different quadrants. Periodontal health group: PD < 3 mm, CAL = 0 mm on all examined teeth.	In periodontitis group and periodontal health group, HbA1c (%, mean ± SD): 4.75 ± 0.47 vs. 4.69 ± 0.59, *p* > 0.05; Glucose (mmol/L, mean ± SD): 4.96 ± 0.59 vs. 4.65 ± 0.43, *p* > 0.05; by one-way ANOVA followed by Fisher’s LSD test.
Hong M (2016) [38]	Cross-sectional study, nationally representative community sample from multi-centers	Periodontitis group and non-periodontitis group: 1005 vs. 2855	Patients with periodontitis mean aged 53.5 ± 0.6 years and 60.9% were males, without periodontitis mean aged 46.9 ± 0.3 years and 45.6% were males	FPG < 126 mg/dL, not self-reported diagnosed with DM and not current use of oral hypoglycemic agents and/or insulin.	Periodontitis: CPI ≥ 3.	In periodontitis group and non-periodontitis group, HbA1c (%, mean ± SD): 5.6 ± 0.02 vs. 5.5 ± 0.0, *p* < 0.05; by one-way ANOVA.
Mishra V (2016) [63]	Case–control study, convenience sample	Chronic periodontitis group and periodontal health group: 14 vs. 14	Patients with periodontitis mean aged 41.71 ± 8.06 years with 8/14 (57.14%) males, healthy controls mean aged 32.43 ± 2.03 years with 3/14 (21.43%) males. Non-smokers	No signs and symptoms of systemic disease.	Periodontitis: AAP 1999. Periodontal health: clinically healthy periodontium.	In periodontitis group and control group, HbA1c (%, mean ± SD): 4.71 ± 0.59 vs. 4.78 ± 0.52, *p* > 0.05; by one-way ANOVA and Tukey’s multiple post hoc procedures.
Vaghani H (2016) [44]	Case–control study, convenience sample	Periodontitis group and non-periodontitis group: 30 vs. 30	35–65 years; non-smokers	Not diagnosed with DM, FBS < 110 mg/dL and without clinical history of DM.	Periodontitis: PD ≥ 5 mm and CAL > 3 mm in five or more teeth. Non-periodontitis group: no BOP, no PD > 3 mm and no CAL.	In periodontitis group and control group, HbA1c (%, mean ± SD): 6.17 ± 0.22 vs. 5.62 ± 0.24, the difference was statistically significant; by ANOVA test.
Acharya AB (2015) [46]	Case–control study, convenience sample	Moderate-severe chronic periodontitis group and periodontal health group: 15 vs. 15	35–55 years; non-smokers; BMI < 30 kg/m^2^	Systemically healthy subjects.	AAP 1999. Periodontitis: at least 4 teeth with PD ≥5 mm, CAL and alveolar BL as evidenced in prescribed radiographs.	In periodontitis group and periodontal health group, HbA1c (%, mean ± SD): 5.78 ± 0.28 vs. 4.86 ± 0.48, *p =* 0.001; RBS (mg/dL, mean ± SD): 103.73 ± 12.47 vs. 114.86 ± 17.76; by one-way ANOVA and Tukey’s multiple post hoc procedures.
Lappin DF (2015) [39]	Cross-sectional study, convenience sample	Periodontitis group and healthy volunteers: 23 vs. 19	Patients with periodontitis mean aged 40 ± 11 years and 46% were males, healthy volunteers mean aged 33 ± 8 years and 63% were males. No history of smoking within the past five years	Healthy volunteers.	Periodontitis: at least two sites with PD and CAL ≥ 5 mm.	In periodontitis group and healthy volunteers, HbA1c (mmol/mol, mean ± SD): 33.3 ± 1.1 vs. 32.2 ± 1.1; blood glucose (mmol/L, mean ± SD): 5.7 ± 0.1 vs.5.6 ± 0.1. The difference between two groups was not compared.
Matic Petrovic S (2015) [55]	Case–control study, convenience sample	Chronic periodontitis group and periodontal health group: 30 vs. 35	Patients with periodontitis mean aged 47.07 ± 10.869 years with 14/30 (40.0%) males, healthy controls mean aged 43.57 ± 3.389 years with 14/35 (28.6%) males	Normal parameters on OGTT and HbA1c < 6.5%.	AAP 1999. Periodontitis: CAL > 1 mm and PD > 3 mm at least at three sites in two quadrants. Control group: PD < 3 mm and CAL = 0 mm.	In periodontitis group and periodontal health group, HbA1c (%, mean ± SD): 4.86 ± 0.635 vs. 4.81 ± 0.623; FPG (mg/dL, mean ± SD): 4.73 ± 0.624 vs. 4.65 ± 0.539. Not reported median. There was no statistical significance between groups using non-parametric test.
Muthu J (2015) [45]	Case–control study, convenience sample	Periodontitis group and control group: 130 vs. 90	96 women and 124 men aged 35–50 years; non-smokers	Nondiabetic patients.	AAP 1999. Periodontitis: ≥ 5 teeth with PD ≥ 5 mm and CAL > 3 mm or radiographic BL, a mSBI ≥ 2 in at least 15% of sites. Control group: No PD ≥ 4 mm, BOP ≤ 15% of tooth sites.	In periodontitis group and control group, HbA1c (%, mean ± SD): 3.40 ± 0.58 vs. 2.23 ± 0.47, *p* < 0.001; by Student’s independent *t*-test.
Srinivasa TS (2015) [54]	Case–control study, convenience sample	Chronic periodontitis group and control group: 20 vs. 20	Patients with periodontitis mean aged 38.9 ± 13.4 years with 12/20 (60.0%) males, healthy controls mean aged 40.1 ± 14.4 years with 10/20 (50.0%) males	No severe systemic diseases.	AAP 1999. Periodontitis: at least five teeth with PD ≥ 5 mm, BOP and CAL > 1 mm on > 5 teeth or radiographic BL. Control group: PD ≤ 4 mm, BOP ≤ 15% and no CAL.	In periodontitis group and control group, HbA1c (%, mean ± SD): 5.66 ± 0.35 vs. 5.17 ± 0.3, *p =* 0.003; by *t*-test.
Corbi SCT (2014) [64]	Case–control study, convenience sample	Chronic periodontitis group and control group: 30 vs. 30	Patients with periodontitis mean aged 45.9 ± 5.9 years with 19/30 (63.3%) males, healthy controls mean aged 39.3 ± 3.6 years with 18/30 (60.0%) males. Never-smokers	Systemically healthy individuals.	AAP 1999. Periodontitis: PD ≥ 6 mm and CAL ≥ 4 mm in ≥4 non-adjacent teeth.	In periodontitis group and control group, HbA1c (%, mean ± SD): 5.1 ± 0.6 vs. 5.4 ± 0.21; fasting glucose (mg/dL, mean ± SD): 90.8 ± 7.3 vs. 85.9 ± 6.5. Not reported median. There was no statistical siginificance between groups using Kruskal–Wallis test and Dunn’s post hoc test.
Gokhale NH (2014) [56]	Case–control study, convenience sample	Chronic periodontitis group and control group: 15 vs. 15	Patients with periodontitis consisted of 3/15 (20.0%) males, healthy controls consisted of 7/15 (46.7%) males. Non-smokers	HbA1c levels <6.5%, and RBS levels <200 mg/dL.	Periodontitis: GI > 1, minimum of three teeth with PD ≥ 5 mm that were positive for BOP and radiographic evidence of BL. Control group: GI ≤ 1, no teeth with PD ≥ 5 mm.	In periodontitis group and control group, HbA1c (%, mean ± SD): 4.75 ± 0.59 vs. 4.75 ± 0.51, *p* > 0.05; RBS (mg/dL, mean ± SD): 127.20 ± 17.37 vs. 126.33 ± 20.36, *p* > 0.05; by one-way ANOVA and Tukey multiple post hoc procedures.
Javed F (2014) [40]	Case–control study, convenience sample	Chronic periodontitis group and control group: 30 vs. 28	Patients with periodontitis mean aged 42.2 ± 1.8 years, healthy controls mean aged 42.7 ± 3.2 years. Only males included. Non-smokers	Self-reported systemically healthy individuals	AAP 1999. Periodontitis: CAL ≥ 3 mm, PD ≥ 5 mm, and marginal BL ≥ 3 mm in >30% of the sites.	In periodontitis group and control group, HbA1c (%, mean ± SD): 4.8 ± 0.5 vs. 4.3 ± 0.2, *p* > 0.05; FPG (mg/dL, mean ± SD): 80.1 ± 3.5 vs. 75.3 ± 2.2, *p* > 0.05; by one-way ANOVA.
Perayil J (2014) [42]	Case–control study, convenience sample	Calculated, periodontitis group and control group: 30 vs. 30	Patients with periodontitis mean aged 45.24 ± 8.50 years with 16/30 (53.3%) males, healthy controls mean aged 40.77 ± 8.29 years with 10/30 (33.3%) males. Non-smokers	FBS levels <110 mg/dL.	AAP 1999. Periodontitis: PD ≥ 5 mm and CAL > 3 mm in ≥5 teeth. Control group: no BOP, no PD > 3 mm, no CAL.	In periodontitis group and control group, HbA1c (%, mean ± SD): 6.08 ± 0.23 vs. 5.38 ± 0.22, *p =* 0.001, by independent sample *t*-tests.
Rajan P (2013) [65]	Case–control study, convenience sample	Chronic periodontitis group and control group: 70 vs. 70	Patients with periodontitis mean aged 45.33 ± 6.64 years with 27/70 (38.6%) males, healthy controls mean aged 43.43 ± 6.57 years with 30/70 (42.9%) males	Previously not diagnosed with DM.	Periodontitis: ≥10 teeth with PD ≥ 5 mm and ≥15% sites with BOP and CAL > 1 mm. Control group: PD <4 mm, BOP at <15% of teeth sites.	In periodontitis group and control group, HbA1c (%, mean ± SD): using kit, 5.51 ± 0.53 vs. 5.44 ± 0.27, *p* > 0.05; in lab, 5.50 ± 0.74 vs. 5.48 ± 0.29, *p* > 0.05; by *t*-test.
Rao Deepika PC (2013) [66]	Case–control study, convenience sample	Severe periodontitis group and control group: 28 (recruited 30 subjects) vs. 30	Patients with periodontitis mean aged 48.82 ± 7.775 years with 19/28 (67.9%) males, healthy controls mean aged 45.93 ± 5.632 years with 12/30 (40.0%) males. Non-smokers	No medical history with DM.	AAP 1999. Severe periodontitis: >30% of the sites with CAL ≥ 5 mm and BOP. Control group: PD ≤ 4 mm, BOP at ≤15% of tooth sites, no CAL.	In periodontitis group and control group, HbA1c (%, median; min and max): 5.8; 5.1 and 6.0 vs. 5.6; 5.1 and 6.0, *p* > 0.05. Subgroup analysis according to BMI: normal-weight subjects: In periodontitis group and control group, HbA1c (%, mean ± SD): 5.68 ± 0.25 vs. 5.62 ± 0.27, *p* > 0.05; overweight subjects: In periodontitis group and control group, HbA1c (%, mean ± SD): 5.89 ± 0.12 vs. 5.65 ± 0.27, *p* = 0.016, by *t*-test.
Zizzi A (2013) [57]	Case–control study, convenience sample	Periodontitis (generalized, severe, chronic) group and control group: 16 vs. 16	Patients with periodontitis mean aged 56.5 ± 1.32 years with 9/16 (56.3%) males, healthy controls mean aged 55 ± 1.76 years with 12/16 (75.0%) males. Non-smokers	HbA1c levels <6.1%, plasma glucose < 100 mg/dL.	AAP 1999. Periodontitis: >30% of sites with >5 mm of CAL. Control group: PD < 3 mm, GI = 0 and CAL < 2 mm.	Median (IQR) of HbA1c (%) in periodontitis group and control group: 5.3 (5.2–5.5) vs. 5 (4.9–5.2), *p* < 0.05; glycemia (mg/dL): 87.5 (78.7–92) vs. 82.5 (76.2–89), *p* > 0.05; by the Kruskal–Wallis test followed by the Mann–Whitney *U*-test.
Saxena RM (2012) [47]	Case–control study, convenience sample	Periodontitis group and control group: 18 vs. 18	Non-smokers	Not diagnosed with DM.	AAP 1999. Periodontitis: ≥5 teeth with PD ≥ 5 mm, and >5 teeth with CAL > 1 mm or radiographic BL and BOP. Control group: PD ≤ 4 mm, BOP at ≤15% of tooth sites, and no CAL.	In periodontitis group and control group, HbA1c (%, mean ± SD): 6.0611 ± 0.0645 vs. 5.7944 ± 0.1830, *p* > 0.05; subgroup analysis according to BMI: normal-weight subjects: in periodontitis group and control group, HbA1c (%, mean): 6.08 vs. 5.78, *p* > 0.05; overweight subjects: in periodontitis group and control group, HbA1c (%, mean): 5.7 vs. 5.87, *p* > 0.05, by *t*-test.
Pan Z (2010) [67]	Case–control study, convenience sample	Chronic periodontitis group and control group: 20 vs. 20	Patients with periodontitis mean aged 43.1 ± 8.9 years with 12/20 (60.0%) males, healthy controls mean aged 29.8 ± 9.2 years with 12/20 (60.0%) males. Non-smokers	Systemically healthy subjects.	AAP 1999. Periodontitis: ≥20 teeth with >30% of measured sites with CAL > 5 mm, BOP at >50% of the proximal sites and alveolar BL > 50% in at least two quadrants. Control group: PD < 3 mm with no CAL, no obvious clinical inflammation, and no BOP.	In periodontitis group and control group, HbA1c (%, mean ± SD): 4.9 ± 0.7 vs. 5.0 ± 0.6, *p* > 0.05. FPG (mg/dL, mean ± SD): 87.2 ± 7.9 vs. 87.8 ± 7.3, *p* > 0.05; by parametric tests.
Wolff RE (2009) [41]	Case–control study, convenience sample	Calculated; periodontitis group and control group: 59 vs. 53	Patients with periodontitis mean aged 51.3 ± 14.8 years with 40/59 (67.8%) males, healthy controls mean aged 50.9 ± 16.3 years with 20/53 (37.7%) males	Not diagnosed with DM.	AAP 1999. Periodontitis: ≥5 teeth with PD ≥ 5 mm and BOP and CAL > 1 mm or radiographic BL. Control group: no PD > 4 mm, BOP at ≤5% of tooth sites, and no periodontal treatment within 6 months.	In periodontitis group and control group, HbA1c (%, mean ± SD): 5.66 ± 0.56 vs. 5.51 ± 0.44, *p* = 0.12,by *t*-test. After adjustments for age, gender, BMI, and current smoking, mean HbA1c was significantly higher in cases than controls (0.21%; 95% CI 0.01% to 0.41%; *p* = 0.046), by multivariate linear regression.
Akalin FA (2008) [68]	Case–control study, convenience sample	Chronic periodontitis group and control group: 17 vs. 17	Patients with periodontitis mean aged 49.35 ± 9.07 years with 9/17 (52.9%) males, healthy controls mean aged 44.12 ± 9.54 years with 8/17 (47.1%) males. Never-smokers	Not having systemic disease and a history of DM in the family.	AAP 1999. Periodontitis: ≥30% periodontal BL and ≥3 teeth with ≥ 5 mm periodontal pockets. Control group: no gingival inflammation, no history of any periodontal disease, with PD of <3 mm, and had good oral hygiene.	In periodontitis group and control group, HbA1c (%, median): 5.4 vs. 5.2, *p* > 0.05; FPG (mg/dL, median): 82 vs. 82, *p* > 0.05; by Kruskal–Wallis test.

* Study design was based on the parts we focused on. AAP 1999: The 1999 International Workshop for a Classification of Periodontal Diseases and Conditions; AAP/EFP: The American Academy of Periodontology and European Federation of Periodontology; ADA: American Diabetes Association; ANOVA: analysis of variance; BL: bone loss; BMI: body mass index; BOP: bleeding on probing; CAL: clinical attachment level; CDC: the Centers for Disease Control and Prevention; CEJ: cemento-enamel junction; CPI: community periodontal index; DM: diabetes mellitus; FBS: fasting blood sugar; FPG: fasting plasma glucose; GI: gingival index; HbA1c: glycated hemoglobin; IQR: interquartile range; KNHANES: Korean National Health and Nutritional Examination Survey; mSBI: modified sulcus bleeding index; OGTT: oral glucose tolerance test; OHI-S: oral hygiene index-simplified; PD: probing depth; PI: plaque index; RBS: random blood sugar; SD: standard deviation

## Data Availability

The data that support the findings of this study are available from the corresponding author upon reasonable request.

## Supplementary Materials

The following supporting information can be downloaded at: https://www.mdpi.com/article/10.3390/healthcare11192649/s1, Figure S1: Funnel plot for investigating publication bias of cross-sectional studies; Figure S2: Funnel plot for investigating publication bias of case–control studies; Figure S3: Stability of the pooled estimation in cross-sectional studies; Figure S4: Stability of the pooled estimation in case–control studies; Table S1: Database search strategy; Table S2: Data collection of details about periodontal examinations, covariates, clinical assessment methods, and normality test; Table S3: The Newcastle–Ottawa Quality Assessment Scale modified for cross-sectional studies (Max. 8 *); Table S4: Quality assessment of included studies (n = 34).

## References

[1] Hajishengallis G.L.J. (2012). Complement and dysbiosis in periodontal disease. Immunobiology.

[2] Spahr A., Divnic-Resnik T. (2022). Impact of health and lifestyle food supplements on periodontal tissues and health. Periodontology 2000.

[3] Peres M.A., Macpherson L.M.D., Weyant R.J., Daly B., Venturelli R., Mathur M.R., Listl S., Celeste R.K., Guarnizo-Herreno C.C., Kearns C. (2019). Oral diseases: A global public health challenge. Lancet.

[4] Diseases G.B.D., Injuries C. (2020). Global burden of 369 diseases and injuries in 204 countries and territories, 1990–2019: A systematic analysis for the Global Burden of Disease Study 2019. Lancet.

[5] Luo L.S., Luan H.H., Jiang J.F., Wu L., Li C., Leng W.D., Zeng X.T. (2022). The spatial and temporal trends of severe periodontitis burden in Asia, 1990–2019: A population-based epidemiological study. J. Periodontol..

[6] Hajishengallis G., Chavakis T. (2021). Local and systemic mechanisms linking periodontal disease and inflammatory comorbidities. Nat. Rev. Immunol..

[7] Genco R.J., Sanz M. (2020). Clinical and public health implications of periodontal and systemic diseases: An overview. Periodontology 2000.

[8] Martinez-Garcia M., Hernandez-Lemus E. (2021). Periodontal Inflammation and Systemic Diseases: An Overview. Front. Physiol..

[9] Bui F.Q., Almeida-da-Silva C.L.C., Huynh B., Trinh A., Liu J., Woodward J., Asadi H., Ojcius D.M. (2019). Association between periodontal pathogens and systemic disease. Biomed. J..

[10] Beck J.D., Papapanou P.N., Philips K.H., Offenbacher S. (2019). Periodontal Medicine: 100 Years of Progress. J. Dent. Res..

[11] Shoelson S.E., Lee J., Goldfine A.B. (2006). Inflammation and insulin resistance. J. Clin. Investig..

[12] Loos B.G., Craandijk J., Hoek F.J., Wertheim-van Dillen P.M., van der Velden U. (2000). Elevation of systemic markers related to cardiovascular diseases in the peripheral blood of periodontitis patients. J. Periodontol..

[13] Tonetti M.S., D’Aiuto F., Nibali L., Donald A., Storry C., Parkar M., Suvan J., Hingorani A.D., Vallance P., Deanfield J. (2007). Treatment of periodontitis and endothelial function. N. Engl. J. Med..

[14] Genco R.J., Genco F.D. (2014). Common risk factors in the management of periodontal and associated systemic diseases: The dental setting and interprofessional collaboration. J. Evid. Based Dent. Pract..

[15] Stanko P., Izakovicova Holla L. (2014). Bidirectional association between diabetes mellitus and inflammatory periodontal disease. A review. Biomed. Pap. Med. Fac. Univ. Palacky. Olomouc. Czechoslov..

[16] Preshaw P.M., Alba A.L., Herrera D., Jepsen S., Konstantinidis A., Makrilakis K., Taylor R. (2012). Periodontitis and diabetes: A two-way relationship. Diabetologia.

[17] Taylor G.W., Burt B.A., Becker M.P., Genco R.J., Shlossman M., Knowler W.C., Pettitt D.J. (1996). Severe periodontitis and risk for poor glycemic control in patients with non-insulin-dependent diabetes mellitus. J. Periodontol..

[18] Grossi S.G., Genco R.J. (1998). Periodontal disease and diabetes mellitus: A two-way relationship. Ann. Periodontol..

[19] Mealey B.L., Ocampo G.L. (2007). Diabetes mellitus and periodontal disease. Periodontology 2000.

[20] Lang N.P., Suvan J.E., Tonetti M.S. (2015). Risk factor assessment tools for the prevention of periodontitis progression a systematic review. J. Clin. Periodontol..

[21] Rapone B., Ferrara E., Corsalini M., Qorri E., Converti I., Lorusso F., Delvecchio M., Gnoni A., Scacco S., Scarano A. (2021). Inflammatory Status and Glycemic Control Level of Patients with Type 2 Diabetes and Periodontitis: A Randomized Clinical Trial. Int. J. Environ. Res. Public Health.

[22] Rapone B., Ferrara E., Corsalini M., Converti I., Grassi F.R., Santacroce L., Topi S., Gnoni A., Scacco S., Scarano A. (2020). The Effect of Gaseous Ozone Therapy in Conjunction with Periodontal Treatment on Glycated Hemoglobin Level in Subjects with Type 2 Diabetes Mellitus: An Unmasked Randomized Controlled Trial. Int. J. Environ. Res. Public. Health.

[23] Faggion C.M., Cullinan M.P., Atieh M. (2016). An overview of systematic reviews on the effectiveness of periodontal treatment to improve glycaemic control. J. Periodontal. Res..

[24] Teshome A., Yitayeh A. (2016). The effect of periodontal therapy on glycemic control and fasting plasma glucose level in type 2 diabetic patients: Systematic review and meta-analysis. BMC Oral. Health.

[25] Stohr J., Barbaresko J., Neuenschwander M., Schlesinger S. (2021). Bidirectional association between periodontal disease and diabetes mellitus: A systematic review and meta-analysis of cohort studies. Sci. Rep..

[26] Nguyen A.T.M., Akhter R., Garde S., Scott C., Twigg S.M., Colagiuri S., Ajwani S., Eberhard J. (2020). The association of periodontal disease with the complications of diabetes mellitus. A Syst. Rev. Diabetes Res. Clin. Pr. Pract..

[27] Kocher T., König J., Borgnakke W.S., Pink C., Meisel P. (2018). Periodontal complications of hyperglycemia/diabetes mellitus: Epidemiologic complexity and clinical challenge. Periodontology 2000.

[28] Moher D., Liberati A., Tetzlaff J., Altman D.G., Group P. (2010). Preferred reporting items for systematic reviews and meta-analyses: The PRISMA statement. Int. J. Surg..

[29] Wells G., Shea B., O’Connell D., Peterson J., Welch V., Losos M., Tugwell P. (2014). The Newcastle-Ottawa Scale (NOS) for Assessing the Quality of Nonrandomised Studies in Meta-Analyses.

[30] Elyasi M., Abreu L.G., Badri P., Saltaji H., Flores-Mir C., Amin M. (2015). Impact of Sense of Coherence on Oral Health Behaviors: A Systematic Review. PLoS ONE.

[31] Ferreira M.C., Dias-Pereira A.C., Branco-de-Almeida L.S., Martins C.C., Paiva S.M. (2017). Impact of periodontal disease on quality of life: A systematic review. J. Periodontal. Res..

[32] Chambrone L., Foz A.M., Guglielmetti M.R., Pannuti C.M., Artese H.P., Feres M., Romito G.A. (2013). Periodontitis and chronic kidney disease: A systematic review of the association of diseases and the effect of periodontal treatment on estimated glomerular filtration rate. J. Clin. Periodontol..

[33] Egger M., Davey Smith G., Schneider M., Minder C. (1997). Bias in meta-analysis detected by a simple, graphical test. BMJ.

[34] Begg C.B., Mazumdar M. (1994). Operating characteristics of a rank correlation test for publication bias. Biometrics.

[35] Yilmaz D., Topcu A.O., Akcay E.U., Altındis M., Gursoy U.K. (2020). Salivary human beta-defensins and cathelicidin levels in relation to periodontitis and type 2 diabetes mellitus. Acta Odontol. Scand..

[36] Matic Petrovic S., Radunovic M., Barac M., Kuzmanovic Pficer J., Pavlica D., Arsic Arsenijevic V., Pucar A. (2019). Subgingival areas as potential reservoirs of different Candida spp in type 2 diabetes patients and healthy subjects. PLoS ONE.

[37] Han K., Park J.-B. (2018). Clinical implication of fasting glucose and systolic/diastolic blood p ressure on the prevalence of periodontitis in non-diabetic and non-hyp ertensive adults using nationally representative data. Exp. Ther. Med..

[38] Hong M., Kim H.Y., Seok H., Yeo C.D., Kim Y.S., Song J.Y., Lee Y.B., Lee D.H., Lee J.I., Lee T.K. (2016). Prevalence and risk factors of periodontitis among adults with or without diabetes mellitus. Korean J. Intern. Med..

[39] Lappin D.F., Robertson D., Hodge P., Treagus D., Awang R.A., Ramage G., Nile C.J. (2015). The Influence of Glycated Hemoglobin on the Cross Susceptibility Between Type 1 Diabetes Mellitus and Periodontal Disease. J. Periodontol..

[40] Javed F., Ahmed H.B., Saeed A., Mehmood A., Bain C. (2014). Whole salivary interleukin-6 and matrix metalloproteinase-8 levels in patients with chronic periodontitis with and without prediabetes. J. Periodontol..

[41] Wolff R.E., Wolff L.F., Michalowicz B.S. (2009). A pilot study of glycosylated hemoglobin levels in periodontitis cases and healthy controls. J. Periodontol..

[42] Perayil J., Suresh N., Fenol A., Vyloppillil R., Bhaskar A., Menon S. (2014). Comparison of glycated hemoglobin levels in individuals without diabetes and with and without periodontitis before and after non-surgical periodontal therapy. J. Periodontol..

[43] Agrawal A.A., Kolte A.P., Kolte R.A., Chari S., Gupta M., Pakhmode R. (2019). Evaluation and comparison of serum vitamin D and calcium levels in periodontally healthy, chronic gingivitis and chronic periodontitis in patients with and without diabetes mellitus—a cross-sectional study. Acta Odontol. Scand..

[44] Vaghani H., Mehta R., Desai K., Duseja S., Mehta T. (2016). Effect of Non-surgical Periodontal Therapy on Glycosylated Haemoglobin Levels in Diabetics and Non-diabetic Healthy Controls with Periodontitis. Adv. Hum. Human. Biol. May Aug..

[45] Muthu J., Muthanandam S., Mahendra J., Namasivayam A., John L., Logaranjini A. (2015). Effect of Nonsurgical Periodontal Therapy on the Glycaemic Control of Nondiabetic Periodontitis Patients: A Clinical Biochemical Study. Oral. Health Prev. Dent..

[46] Acharya A.B., Thakur S., Muddapur M.V. (2015). Effect of scaling and root planing on serum interleukin-10 levels and glycemic control in chronic periodontitis and type 2 diabetes mellitus. J. Ind. Soc. Periodontol..

[47] Saxena R.M., Deepika P.C. (2012). Comparison of glycosylated hemoglobin levels in periodontitis patients and healthy controls: A pilot study in Indian population. Ind. J. Dent. Res. Off. Publ. Ind. Soc. Dent. Res..

[48] Doğan Ş.B., Ballı U., Dede F., Sertoğlu E., Tazegül K. (2016). Chemerin as a Novel Crevicular Fluid Marker of Patients With Periodontitis and Type 2 Diabetes Mellitus. J. Periodontol..

[49] Bozkurt Doğan Ş., Öngöz Dede F., Ballı U., Sertoğlu E. (2016). Levels of vaspin and omentin-1 in gingival crevicular fluid as potential markers of inflammation in patients with chronic periodontitis and type 2 diabetes mellitus. J. Oral. Sci..

[50] George A.K., Narayan V., Kurian N., Joseph A.E., Anil S. (2021). A pilot study on glycemia and insulin resistance in patients with severe periodontitis. J. Ind. Soc. Periodontol..

[51] Zainab A., Ashish N., Ragnath V. (2019). Salivary Levels of Antimicrobial Peptides in Chronic Periodontitis Patients with Type 2 Diabetes. J. Int. Acad. Periodontol..

[52] Acharya A.B., Thakur S., Muddapur M.V., Kulkarni R.D. (2018). Systemic Cytokines in Type 2 Diabetes Mellitus and Chronic Periodontitis. Curr. Diabetes Rev..

[53] Grdović N., Rajić J., Petrović S.M., Dinić S., Uskoković A., Mihailović M., Jovanović J.A., Tolić A., Pucar A., Milašin J. (2016). Association of CXCL12 gene promoter methylation with periodontitis in patients with diabetes mellitus type 2. Arch. Oral. Biol..

[54] Srinivasa T.S., Agrawal P., Goyal P., Farista S., Sowmya N.K., Deonani S. (2015). Comparative clinical evaluation of glycosylated haemoglobin level in healthy and chronic periodontitis patients: A chairside diagnostic method. Ind. J. Dent. Res. Off. Publ. Ind. Soc. Dent. Res..

[55] Matić Petrović S., Cimbaljević M., Radunović M., Kuzmanović Pfićer J., Jotić A., Pucar A. (2015). Detection and sampling methods for isolation of Candida spp. from oral cavities in diabetics and non-diabetics. Braz. Oral. Res..

[56] Gokhale N.H., Acharya A.B., Patil V.S., Trivedi D.J., Setty S., Thakur S.L. (2014). Resistin levels in gingival crevicular fluid of patients with chronic periodontitis and type 2 diabetes mellitus. J. Periodontol..

[57] Zizzi A., Tirabassi G., Aspriello S.D., Piemontese M., Rubini C., Lucarini G. (2013). Gingival advanced glycation end-products in diabetes mellitus-associated chronic periodontitis: An immunohistochemical study. J. Periodontal. Res..

[58] Gd G.S.G., Sudhakar U., Raghavan A., Narayan K.V. (2023). Effects of Non-surgical Periodontal Therapy on Saliva and Gingival Crevicular Fluid Levels of Chemerin in Periodontitis Subjects with and without Type 2 Diabetes Mellitus. Cureus.

[59] Mahendra J., Palathingal P., Mahendra L., Alzahrani K.J., Banjer H.J., Alsharif K.F., Halawani I.F., Muralidharan J., Annamalai P.T., Verma S.S. (2022). Impact of Red Complex Bacteria and TNF-α Levels on the Diabetic and Renal Status of Chronic Kidney Disease Patients in the Presence and Absence of Periodontitis. Biology.

[60] Akram Z., Alqahtani F., Alqahtani M., Al-Kheraif A.A., Javed F. (2020). Levels of advanced glycation end products in gingival crevicular fluid of chronic periodontitis patients with and without type-2 diabetes mellitus. J. Periodontol..

[61] Altıngöz S.M., Kurgan Ş., Önder C., Serdar M.A., Ünlütürk U., Uyanık M., Başkal N., Tatakis D.N., Günhan M. (2020). Salivary and serum oxidative stress biomarkers and advanced glycation end products in periodontitis patients with or without diabetes: A cross-sectional study. J. Periodontol..

[62] Suresh R., Jayachandran P., Fenol A., Biswas R., Krishnan S., Kumar K.A., Divakar D.D., Vellappally S. (2019). Effect of Non-Surgical Periodontal Therapy on the Serum Sialic Acid Levels in Diabetic Patients with Periodontitis. Acta Med..

[63] Mishra V., Shettar L., Bajaj M., Math A.S., Thakur S.L. (2016). Interlinking Periodontitis and Type 2 Diabetes Mellitus by Assessment of Crevicular Visfatin Levels in Health and in Disease before and after Initial Periodontal Therapy. J. Clin. Diagn. Res..

[64] Corbi S.C., Bastos A.S., Orrico S.R., Secolin R., Dos Santos R.A., Takahashi C.S., Scarel-Caminaga R.M. (2014). Elevated micronucleus frequency in patients with type 2 diabetes, dyslipidemia and periodontitis. Mutagenesis.

[65] Rajan P., Nera M., Pavalura A.K., Medandrao N., Kumar S.C. (2013). Comparison of glycosylated hemoglobin (HbA1C) levels in patients with chronic periodontitis and healthy controls. Dent. Res. J..

[66] Rao Deepika P.C., Saxena R.M. (2013). Comparison of glycosylated hemoglobin levels in severe periodontitis patients and healthy controls: A study in an Indian population. Quintessence Int..

[67] Pan Z., Guzeldemir E., Toygar H.U., Bal N., Bulut S. (2010). Nitric oxide synthase in gingival tissues of patients with chronic periodontitis and with and without diabetes. J. Periodontol..

[68] Akalin F.A., Işiksal E., Baltacioğlu E., Renda N., Karabulut E. (2008). Superoxide dismutase activity in gingiva in type-2 diabetes mellitus patients with chronic periodontitis. Arch. Oral. Biol..

[69] Needleman I., Warnakulasuriya S., Sutherland G., Bornstein M.M., Casals E., Dietrich T., Suvan J. (2006). Evaluation of tobacco use cessation (TUC) counselling in the dental office. Oral. Health Prev. Dent..

[70] Sackett D.L. (1993). Rules of evidence and clinical recommendations for the management of patients. Can. J. Cardiol..

[71] Selvin E., Crainiceanu C.M., Brancati F.L., Coresh J. (2007). Short-term variability in measures of glycemia and implications for th e classification of diabetes. Arch. Intern. Med..

[72] Gillett M.J. (2009). International Expert Committee report on the role of the A1c assay in the diagnosis of diabetes: *Diabetes Care*
**2009**, *32*, 1327–1334. Clin. Biochem. Rev..

[73] Little R.R., Rohlfing C.L., Sacks D.B. (2011). Status of hemoglobin A1c measurement and goals for improvement: From chaos to order for improving diabetes care. Clin. Chem..

[74] Association A.D. (2020). Classification and Diagnosis of Diabetes: Standards of Medical Care in Diabetes-2020. Diabetes Care.

[75] Schulz K.F., Grimes D.A. (2002). Case-control studies: Research in reverse. Lancet.

[76] Zelig R., Samavat H., Duda P., Singer S.R., Feldman C., LaSalle P., Muhammad E., Touger-Decker R. (2023). Screening for diabetes risk using the diabetes risk test and point-of-care hemoglobin A1C values in adults seen in a dental clinic. Quintessence Int..

