# Supplementary material for: Association between Periodontitis and HbA1c Levels in Non-Diabetic Patients: A Systematic Review and Meta-Analysis
Source: Healthcare (Basel). 2023 Sep 28;11(19):2649. doi: 10.3390/healthcare11192649 (PMC10572398; doi:10.3390/healthcare11192649)
Supplement: Supplementary file 1 [file healthcare-11-02649-s001.zip › Table S1. Search Term.pdf]

Table S1. Database search strategy

Database	Search strategy
EMBASE<br>Embase 1947 to 2021 May 07	(Prediabetic State/ or pre-diabetes.mp. or prediabetes.mp. or pre-diabetic.mp. or prediabetic.mp. or pre-diabetics.mp. or prediabetics.mp. or (non-diabetes or nondiabetes or non-diabetic or nondiabetic or non-diabetics or nondiabetics).mp. or systemically healthy.mp. or without diabetes.mp.) and (Glycated Hemoglobin A/ or glycosylated hemoglobin.mp. or HbA1c.mp.) and (periodontitis.mp. or Aggressive Periodontitis/ or Chronic Periodontitis/ or Periodontitis/ or (periodontal.mp. or Periodontal Diseases/ or Periodontal Pocket/) or (oral health.mp. or Oral health/) or (Tooth Loss/ or tooth loss.mp.) or (Mouth, Edentulous/ or edentulism.mp.))
MEDLINE<br>Ovid MEDLINE(R) Epub Ahead of Print, In-Process, In-Data-Review & Other Non-Indexed Citations, Daily and Versions(R) <1946 to May 07, 2021>	(Prediabetic State/ or pre-diabetes.mp. or prediabetes.mp. or pre-diabetic.mp. or prediabetic.mp. or pre-diabetics.mp. or prediabetics.mp. or (non-diabetes or nondiabetes or non-diabetic or nondiabetic or non-diabetics or nondiabetics).mp. or systemically healthy.mp. or without diabetes.mp.) and (Glycated Hemoglobin A/ or glycosylated hemoglobin.mp. or HbA1c.mp.) and (periodontitis.mp. or Aggressive Periodontitis/ or Chronic Periodontitis/ or Periodontitis/ or (periodontal.mp. or Periodontal Diseases/ or Periodontal Pocket/) or (oral health.mp. or Oral health/) or (Tooth Loss/ or tooth loss.mp.) or (Mouth, Edentulous/ or edentulism.mp.))
Web of Science (1987-2021)	("periodontitis" OR "periodontal" OR "oral health" OR "tooth loss" OR "edentulism" OR "mouth, edentulous") AND ("prediabetic state" or "pre-diabetes" or "prediabetes" or "pre-diabetic" or "prediabetic" or "pre-diabetics" or

	"prediabetics" or "non-diabetes" or "nondiabetes" or "non-diabetic" or "nondiabetic" or "non-diabetics" or "nondiabetics" or "systemically healthy" or "without diabetes") AND ("Glycated Hemoglobin A" or "glycosylated hemoglobin" or "HbA1c")
Cochrane Library	((MeSH descriptor: [Periodontitis] explode all trees) OR (MeSH descriptor: [Periodontal Diseases] explode all trees) OR (MeSH descriptor: [Oral Health] explode all trees) OR (MeSH descriptor: [Tooth Loss] explode all trees) OR (MeSH descriptor: [Periodontal Attachment Loss] explode all trees) OR ((periodontal) OR (Periodontitis) OR (oral health) OR (tooth loss) OR (edentulism) (Word variations have been searched))) AND ((MeSH descriptor: [Prediabetic State] explode all trees) OR ((pre-diabetes) OR (prediabetes) OR (pre-diabetic) OR (prediabetic) OR (pre-diabetics) (Word variations have been searched)) OR ((prediabetics) OR (non-diabetes) OR (nondiabetes) OR (non-diabetic) OR (nondiabetic) (Word variations have been searched)) OR ((non-diabetics) OR (nondiabetics) OR (systemically healthy) OR (without diabetes) OR (prediabetic state) (Word variations have been searched))) AND ((MeSH descriptor: [Glycated Hemoglobin A] explode all trees) OR ((Glycated Hemoglobin A) OR (glycosylated hemoglobin) OR (HbA1c) (Word variations have been searched)))
PubMed	(((((Periodontitis[MeSH Terms]) OR (Periodontal Diseases[MeSH Terms])) OR (Oral Health[MeSH Terms])) OR (Tooth Loss[MeSH Terms])) OR (Periodontal Attachment Loss[MeSH Terms]) OR (periodontal) OR (edentulism)) AND ((Prediabetic State[MeSH Terms]) OR (((((pre-diabetes) OR (prediabetes)) OR (pre-diabetic))

	OR (prediabetic)) OR (pre-diabetics)) OR (prediabetics) OR (((((non-diabetes) OR (nondiabetes)) OR (non-diabetic)) OR (nondiabetic)) OR (non-diabetics)) OR (nondiabetics) OR (systemically healthy)) AND ((Glycated Hemoglobin A[MeSH Terms]) OR (glycosylated hemoglobin) OR (HbA1c))
Open Grey	("Periodontitis" OR "Periodontal Diseases" OR "Oral Health" OR "Tooth Loss" OR "Periodontal Attachment Loss" OR "periodontal" OR "edentulism") AND ("Prediabetic State" OR "pre-diabetes" OR "prediabetes" OR "pre-diabetic" OR "prediabetic" OR "pre-diabetics" OR "prediabetics" OR "non-diabetes" OR "nondiabetes" OR "non-diabetic" OR "nondiabetic" OR "non-diabetics" OR "nondiabetics" OR "systemically healthy" OR "without diabetes") AND ("Glycated Hemoglobin A" OR "glycosylated hemoglobin" OR "HbA1c")
