# Supplementary material for: Association between Periodontitis and HbA1c Levels in Non-Diabetic Patients: A Systematic Review and Meta-Analysis
Source: Healthcare (Basel). 2023 Sep 28;11(19):2649. doi: 10.3390/healthcare11192649 (PMC10572398; doi:10.3390/healthcare11192649)
Supplement: Supplementary file 1 [file healthcare-11-02649-s001.zip › Table S2. Data collection.pdf]

Table S2. Data collection of details about periodontal examinations, covariates, clinical assessment methods, and normality test.

First author (publication year)	Origin of subjects	Country (city)	Other information of periodontitis (examination, teeth number, previous periodontal treatment)	Covariates (e,g.,age, gender, BMI, smoking habits)	Clinical assessment methods (blinding; training and calibration)	Normality test	Title
Gomathi GD (2023)	Outpatient clinic of the department of periodontics and implantology, Thai Moogambigai Dental College and Hospital, D. MGR Educational and Research Institute, Chennai.	India (Chennai)	Without any therapy in the past six months	Not mention	Not mention	No	Effects of Non-surgical Periodontal Therapy on Saliva and Gingival Crevicular Fluid Levels of Chemerin in Periodontitis Subjects With and Without Type 2 Diabetes Mellitus
Mahendra J (2022)	Controls from a general hospital of PSM College of Dental Science and Research, Thrissur; cases from PSM Dental College of Science, Kerala, between January 2019 and February 2020.	India (Thrissur)	At least 12 natural teeth; without periodontal treatment in the past 6 months	Gender, age, body mass index and socioeconomic status in periodontitis group and control group were present but no statistical comparison.	Blinded; one trained investigator	Yes	Impact of Red Complex Bacteria and TNF-α Levels on the Diabetic and Renal Status of Chronic Kidney Disease Patients in the Presence and Absence of Periodontitis
George AK (2021)	Department of Periodontics, Pushpagiri College of Dental Sciences, Pushpagiri Institute of Medical Sciences and Research Centre, Thiruvalla, Kerala, India, between June 2018 to June 2019.	India (Kerala)	Six sites per-tooth; full mouth; at least 20 natural teeth	Age, gender and body mass index in Periodontitis group and periodontal health group p>0.05.	A single trained periodontist	No	A pilot study on glycemia and insulin resistance in patients with severe periodontitis
Akram Z (2020)	Not reported	Saudi Arabia (Riyadh)	Six sites per-tooth; full mouth, dentate	The body mass index in periodontitis group and periodontal health group $p<0.05$; gender and age in periodontitis group and periodontal health group $p>0.05$.	Blinded; a trained and calibrated examiner (kappa value 0.92)	Yes	Levels of advanced glycation end products in gingival crevicular fluid of chronic periodontitis patients with and without type-2 diabetes mellitus

Altingoz SM (2020)	The Department of Periodontology, School of Dentistry, and the Department of Endocrinology, School of Medicine, at Ankara University between 03/2011 and 03/2013.	Turkey (Ankara)	Six sites per-tooth at all teeth present, except third molars; at least 16 teeth remained; without non-surgical periodontal therapy in the previous 6 months and surgical periodontal therapy in the previous 12 months	Gender, age, smoking and body mass index in periodontitis group and periodontal health group $p>0.05$.	One trained and calibrated examiner	Yes	Salivary and serum oxidative stress biomarkers and advanced glycation end products in periodontitis patients with or without diabetes: A cross-sectional study
Yilmaz D (2020)	The Periodontology Department of Faculty of Dentistry, Sakarya University and Internal Medicine Department of Faculty of Medicine, Sakarya University between March 2018 and January 2019.	Turkey (Sakarya)	Six sites per-tooth; full mouth excluded third molar; without periodontal treatment in the past 6 months	Gender and smoking in Periodontitis group and periodontal health group $p>0.05$.	One calibrated specialist (Kappa value 0.91)	Yes	Salivary human beta-defensins and cathelicidin levels in relation to periodontitis and type 2 diabetes mellitus
Agrawal AA (2019)	The out-patient department, Department of Periodontics and Implantology, VSPM Dental College and Research Centre, Nagpur, India.	India (Nagpur)	Six sites per tooth in all present teeth excluding third molars; without periodontal treatment in the past 6 months	Age in periodontitis group and periodontal health group were shown but no statistical comparison.	A single examiner (ICC were between 0.92 and 0.99 in the groups)	No	Evaluation and comparison of serum vitamin D and calcium levels in periodontally healthy, chronic gingivitis and chronic periodontitis in patients with and without diabetes mellitus - a cross-sectional study
Matic Petrovic S (2019)	The Department of Periodontology and Oral Medicine, School of Dental Medicine, University of Belgrade from February 2015 to April 2017	Serbia (Belgrade)	Six sites per-tooth; full mouth except third molars; more than 14 teeth remained; without periodontal treatment in the past 1.5 years	Gender, age and smoking in periodontitis group and periodontal health group $p>0.05$.	Two experienced and calibrated doctors (Kappa value 0.5-0.6)	No	Subgingival areas as potential reservoirs of different Candida spp in type 2 diabetes patients and healthy subjects
Suresh R (2019)	Department of Periodontics, Amrita School of Dentistry, Amrita vishwa vidhyapeetham, Ponekara, Edapally, Cochin, India.	India (Cochin)	Six sites per tooth in all teeth, excluding third molars; at least 20 natural teeth; without periodontal treatment in the past 6 months	The body mass index in periodontitis group and periodontal health group $p<0.05$; gender and age in periodontitis group and periodontal health group $p>0.05$.	A single examiner	No	Effect of Non-Surgical Periodontal Therapy on the Serum Sialic Acid Levels in Diabetic Patients with Periodontitis

Zainab AJAA (2019)	India	India (Karnataka)	Full mouth excluding third molars; at least 20 functional teeth; without professional periodontal treatment in the past 6 months	Gender matched in periodontitis group and periodontal health group ; age in periodontitis group and periodontal health group *p*<0.05.	A single, calibrated examiner (κ-light test, agreement >0.85)	No	Salivary Levels of Antimicrobial Peptides in Chronic Periodontitis Patients with Type 2 Diabetes
Acharya AB (2018)	Volunteers (Professionals, administrative staff, health care workers), and patients visited outpatient departments of their associated hospitals.	India (Karnataka)	Dentate; without periodontal treatment in the past 90 days	Age in periodontitis group and periodontal health group *p*>0.05.	Not mention	No	Systemic Cytokines in Type 2 Diabetes Mellitus and Chronic Periodontitis
Han K (2018)	The KNHANES from 2013 to 2015.	Korea	Six sites per-tooth; at least 2 teeth remained	Gender, age, smoking and body mass index in periodontitis group and periodontal health group *p*<0.05.	Trained dental practitioners	No	Clinical implication of fasting glucose and systolic/diastolic blood pressure on the prevalence of periodontitis in non-diabetic and non-hypertensive adults using nationally representative data
Dogan SB (2016a)	The Department of Periodontology, Faculty of Dentistry, Bülent Ecevit University, Zonguldak, Turkey, between July 2014 and May 2015.	Turkey (Ankara)	Six sites per-tooth; full mouth excluding third molars; at least 20 teeth; without non-surgical periodontal therapy in the previous 6 months and surgical periodontal therapy in the previous year	Gender, age and body mass index in periodontitis group and periodontal health group p>0.05.	A single calibrated examiner (similarity >90% at the millimeter level)	Yes	Levels of vaspin and omentin-1 in gingival crevicular fluid as potential markers of inflammation in patients with chronic periodontitis and type 2 diabetes mellitus
Dogan SB (2016b)	The Department of Peridontology, Faculty of Dentristry, Bülent Ecevit University between the dates of July 2014 and May 2015.	Turkey (Zonguldak)	Six sites per-tooth; full mouth excluding third molars; at least 20 teeth (not including third molars); without non-surgical periodontal therapy in the previous 6 months and surgical periodontal therapy in the previous 12 months	Gender, age and body mass index in periodontitis group and periodontal health group p>0.05.	Blinded; a single calibrated examiner (similarity >90% at the millimeter level)	Yes	Chemerin as a Novel Crevicular Fluid Marker of Patients With Periodontitis and Type 2 Diabetes Mellitus
Grdovic N (2016)	Caucasian subjects of Serbian nationality between March 2013 and January 2015	Serbia (Belgrade)	Six sites per-tooth; full mouth; more than 14 teeth remained; without periodontal treatment in the past 1.5 years	Gender and smoking in periodontitis group and periodontal health group *p*>0.05; age in periodontitis group and periodontal health group *p*<0.05.	Two experienced and calibrated doctors (with a degree of agreement within 1mm in 95%)	Yes	Association of CXCL12 gene promoter methylation with periodontitis in patients with diabetes mellitus type 2

Hong M (2016)	Data derived from the fifth KNHANES, conducted in 2012 by the Korea CDC.	Korea	The eight molars and the upper right and lower left central incisors were examined for each sextant	Gender, age, smoking and body mass index in Periodontitis group and periodontal health group *p*<0.05.	Not mention	No	Prevalence and risk factors of periodontitis among adults with or without diabetes mellitus
Mishra V (2016)	The Department of Periodontics and Oral Implantology, SDM College of Dental Sciences and Hospital, Dharwad, Karanataka, India.	India (Telangana)	At least 20 natural teeth; without periodontal treatment in the past 6 months	Body mass index in periodontitis group and periodontal health group *p*>0.05.	Not mention	No	Interlinking periodontitis and type 2 diabetes mellitus by assessment of crevicular visfatin levels in health and in disease before and after initial periodontal therapy
Vaghani H (2016)	The Department of Periodontics, Karnavati School of Dentistry, Gujarat, India	India (Gujarat)	At least 20 teeth; without periodontal treatment in the past 6 months	Body mass index in periodontitis group and periodontal health group was present but no statistical comparison.	Not mention	No	Effect of Non-surgical Periodontal Therapy on Glycosylated Haemoglobin Levels in Diabetics and Non-diabetic Healthy Controls with Periodontitis
Acharya AB (2015)	The Department of Periodontics and the Department of General Medicine of the associated hospital	India (Karnataka)	At least 20 natural teeth; without nonsurgical therapy for CPD in the past 3 months	Not mention	Not mention	Yes	Effect of scaling and root planing on serum interleukin-10 levels and glycemic control in chronic periodontitis and type 2 diabetes mellitus
Lappin DF (2015)	Diabetic out-patient clinics at Glasgow Royal Infirmary, Stobhill Hospital, and Glasgow Dental Hospital and School (all Glasgow, UK) between 2007 and 2010.	UK (Glasgow)	Six sites per-tooth; full mouth; at least 20 teeth remained	Age in periodontitis group and periodontal health group *p*>0.05.	Not mention	No	The Influence of Glycated Hemoglobin on the Cross Susceptibility Between Type 1 Diabetes Mellitus and Periodontal Disease
Matic Petrovic S (2015)	Not reported	Serbia (Belgrade)	Six sites per-tooth; full mouth; without periodontal treatment in the past 1.5 years	Gender, age and smoking in periodontitis group and periodontal health group *p*>0.05.	Two calibrated doctors	No	Detection and sampling methods for isolation of Candida spp. from oral cavities in diabetics and non-diabetics
Muthu J (2015)	The patient pool of the Department of Periodontology, Meenakshi Ammal Dental College and Hospital, Chennai, India.	India (Chennai)	At least 10 teeth; without periodontal treatment in the past 6 months	Body mass index in periodontitis group and periodontal health group *p*>0.05.	One calibrated examiner	No	Effect of Nonsurgical Periodontal Therapy on the Glycaemic Control of Nondiabetic Periodontitis Patients: A Clinical Biochemical Study

Srinivasa TS (2015)	The Out Patient Department of Periodontics, Rungta College of Dental Sciences and Research, Kohka-Kurud, Bhilai, Chhattisgarh.	India(Karnataka)	Not mention	Age in periodontitis group and control group *p*=0.8; gender in periodontitis group and control group *p*=0.65.	Not mention	No	Comparative clinical evaluation of glycosylated haemoglobin level in healthy and chronic periodontitis patients: A chairside diagnostic method
Corbi SCT (2014)	The Araraquara School of Dentistry, Universidade Estadual Paulista, Brazil	Brazil (Araraquara)	Six sites per-tooth; at least 15 teeth; without periodontal treatment or surgery in the past 6 months	Gender and body mass index in Periodontitis group and periodontal health group *p*>0.05; age in Periodontitis group and periodontal health group *p*<0.05.	One single trained calibrated examiner (Kappa value 0.89)	Yes	Elevated micronucleus frequency in patients with type 2 diabetes, dyslipidemia and periodontitis
Gokhale NH (2014)	The outpatient departments of the Department of Periodontics and Oral Implantology, and the Department of General Medicine, Shri Dharmasthala Manjunateshwara College of Dental Sciences and Hospital in Dharwad, India, between May 2012 and July 2012	India (Karnataka)	At least 20 teeth	Body mass index in periodontitis group and health control group *p*= 0.6867.	A single examiner	Yes	Resistin levels in gingival crevicular fluid of patients with chronic periodontitis and type 2 diabetes mellitus
Javed F (2014)	Periodontitis group: Males from the Department of Dentistry of Jinnah Postgraduate Medical Center, Karachi, Pakistan. Healthy individuals: Males from a residential area. Study conducted from February to June 2013.	Pakistan (Karachi)	Six sites per-tooth; full mouth excluded third molar; dentate; without periodontal treatment in the past 6 months	Gender and age in periodontitis group and periodontal health group *p*>0.05, family history of diabetes in periodontitis group and periodontal health group *p*<0.05.	Blind; a trained and calibrated investigator (Kappa value 0.78)	No	Whole Salivary Interleukin-6 and Matrix Metalloproteinase-8 Levels in Patients With Chronic Periodontitis With and Without Prediabetes
Perayil J (2014)	The Outpatient Department of	India (Kochi)	Six sites per-tooth; full mouth excluded third molar; more than 20 teeth remained; without	Gender and body mass index in periodontitis group and periodontal health group	One trained periodontist	No	Comparison of glycated hemoglobin levels in individuals without diabetes and with and

	Periodontics, Amrita School of Dentistry.		periodontal treatment in the past 6 months	*p*>0.05; age in periodontitis group and periodontal health group *p*<0.05.			without periodontitis before and after non-surgical periodontal therapy
Rajan P (2013)	The outpatient department of Periodontics, St. Joseph Dental College, Eluru, Andra Pradesh, India.	India (Eluru)	More than 10 teeth; without periodontal treatment in the past 6 months	Age and gender in periodontitis group and control group *p*>0.05; body mass index in periodontitis group and control group *p*<0.05.	One examiner	No	Comparison of glycosylated hemoglobin (HbA1C) levels in patients with chronic periodontitis and healthy controls
Rao Deepika PC (2013)	The Department of Periodontology, JSS Dental College and Hospital over a period of 3 months.	India (Karnataka)	Four sites per-tooth; more than 15 teeth; without periodontal treatment in the past 6 months	Age and body mass index in periodontitis group and control group *p*>0.05; gender in periodontitis group and periodontal health group *p*<0.05.	Two trained examiners (Kappa > 0.8)	No	Comparison of HbA1c levels in severe periodontitis patients and healthy controls: A study in an Indian population
Zizzi A (2013)	The Division of Periodontology, Department of Odontostomatological and Specialized Clinical Sciences of the Polytechnic Universityof Marche, Ancona, Italy. Study conducted between 2005-2011.	Italy (Ancona)	Six sites per teeth for the whole mouth excluding third molars; at least 20 teeth; without periodontal treatment in the past 2 years	Body mass index in periodontitis group and periodontal health group *p*<0.05; age in periodontitis group and health control group *p*>0.05.	A single examiner	Yes	Gingival advanced glycation end-products in diabetes mellitus-associated chronic periodontitis: an immunohistochemical study
Saxena RM (2012)	The Department of Periodontics, JSS Dental College and Hospital, a Constituent College of JSS University, Mysore, India, over a period of 2 months.	India (Mysore)	Four sites per-tooth; more than 10 natural teeth; no periodontal treatment undertaken within the previous 6 months	Body mass index in periodontitis group and health control group *p*>0.05.	Not mention	No	Comparison of glycosylated hemoglobin levels in periodontitis patients and healthy controls: A pilot study in Indian population
Pan Z (2010)	The Clinics, Adana Medical and Research Center, Baskent University, between September 2007 and August 2008.	Turkey (Ankara)	Six sites per tooth; full mouth; more than 20 teeth; without periodontal treatment in the past 6 months	Not mention	Not mention	Yes	Nitric Oxide Synthase in Gingival Tissues of Patients With Chronic Periodontitis and With and Without Diabetes
Wolff RE (2009)	The University of Minnesota School of Dentistry clinics	US (Minnesota)	Six sites per-tooth; full mouth; At least 10 teeth remained; without periodontal treatment in the past 6 months	Age in periodontitis group and periodontal health group *p*>0.05; gender, smoking and body mass index, in	One board-certified periodontist	No	A Pilot Study of Glycosylated Hemoglobin Levels in Periodontitis Cases and Healthy Controls

	between June and August 2008.			periodontitis group and periodontal health group *p*<0.05.			
Akalin FA (2008)	Not reported	Turkey (Ankara)	Full mouth periapical radiographs; at least 15 teeth	Not mention	A single examiner	Yes	Superoxide dismutase activity in gingiva in type-2 diabetes mellitus patients with chronic periodontitis
