# Supplementary material for: Association between Periodontitis and HbA1c Levels in Non-Diabetic Patients: A Systematic Review and Meta-Analysis
Source: Healthcare (Basel). 2023 Sep 28;11(19):2649. doi: 10.3390/healthcare11192649 (PMC10572398; doi:10.3390/healthcare11192649)
Supplement: Supplementary file 1 [file healthcare-11-02649-s001.zip › Table S3. Modified NOS for cross-sectional studies.pdf]

Table S3. The Newcastle-Ottawa Quality Assessment Scale modified for cross-sectional studies (Max. 8*)

<u>Note</u>: A maximum of one star (*) is awarded for each numbered item.

SELECTION (max. 4*)

1) Representativeness of the sample

a) Truly representative of the average conditions in the community *

b) Somewhat representative of the average conditions in the community *

c) Selected group of the average conditions (eg. more than 60 years old, women)

d) No description of the derivation of the sample

2) Sample size[§]

a) Justified and satisfactory *

b) Not justified or satisfactory

[§] The sample size is estimated using G*Power 3.1.9.7 (t test-means). For small, medium, and large effect size, the d is estimated as 0.2, 0.5 and 0.8, respectively. Therefore, at least 26, 64 or 394 per group is needed to detect the different effect size by a 5% significance level tested with 80% power.

3) Ascertainment of exposure

a) Related parameters assessment*

b) Record linkage

c) Self report

d) No description

4) Non-response rate

a) Comparability between respondents and non-respondents characteristics is established, and the response rate is descripted.*

b) No description of the response rate or the characteristics of the responders and the non-responders.

COMPARABILITY (max. 2*)

1) The subjects in different outcome groups are comparable, based on the study design or analysis. Confounding factors are controlled.

a) The study controls for the most important factor (smoking or BMI) *

b) The study controls for any additional factors (eg. age, gender) *

OUTCOME (max. 2*)

1) Assessment of outcome

a) Related parameters assessment*

b) Record linkage

c) Self report

d) No description

2) Statistical analysis

a) The statistical analysis used to analyze the data is clearly described and the measurement of the association is presented, including mean differences ± standard deviation and the probability level (*p* value).*

b) The statistical analysis used to analyze the data is not described or incomplete.
