# Supplementary material for: Association between Periodontitis and HbA1c Levels in Non-Diabetic Patients: A Systematic Review and Meta-Analysis
Source: Healthcare (Basel). 2023 Sep 28;11(19):2649. doi: 10.3390/healthcare11192649 (PMC10572398; doi:10.3390/healthcare11192649)
Supplement: Supplementary file 1 [file healthcare-11-02649-s001.zip › Table S4. Quality assessment.pdf]

Table S4. Quality assessment of included studies (n = 34)

**Cross-sectional studies**	**Selection**	**Comparability**	**Outcome**	**Max 8 ***	**Max 100%**
Yilmaz D (2020)	*	* *	*	4/8	50.0%
Matic Petrovic S (2019)	*	* *	* *	5/8	62.5%
Han K (2018)	* * *	* *	* *	7/8	87.5%
Hong M (2016)	* * *	* *	* *	7/8	87.5%
Lappin DF (2015)	*	*	*	3/8	37.5%

**Case-control studies**	**Selection**	**Comparability**	**Exposure**	**Max 9 ***	**Max 100%**
Gomathi GD (2023)	* *		* *	4/9	44.4%
Mahendra J (2022)	* * *	*	* *	6/9	66.7%
George AK (2021)	* * *	* *	* *	7/9	77.8%
Akram Z (2020)	*	*	* *	4/9	44.4%
Altingoz (2020)	* *		* *	4/9	44.4%
Agrawal AA (2019)	*	*	* *	4/9	44.4%
Suresh R (2019)	* *	*	* *	5/9	55.6%
Zainab AJAA (2019)	* *	* *	* *	6/9	66.7%
Acharya AB (2018)	*	*	* *	4/9	44.4%
Dogan SB (2016a)	* * *	*	* *	6/9	66.7%
Dogan SB (2016b)	* * *	*	* *	6/9	66.7%
Grdovic N (2016)	* * *	*	* *	6/9	66.7%
Mishra V (2016)	*	*	* *	4/9	44.4%
Vaghani H (2016)	* *		* *	4/9	44.4%
Acharya AB (2015)	*		* *	3/9	33.3%
Matic Petrovic S (2015)	* *		* *	4/9	44.4%
Muthu J (2015)	*	*	* *	4/9	44.4%
Srinivasa TS (2015)	* * *		* *	5/9	55.6%
Corbi SCT (2014)	*	*	* *	4/9	44.4%
Gokhale NH (2014)	* *	*	* *	5/9	55.6%
Javed F (2014)	* *	* *	* *	6/9	66.7%
Perayil J (2014)	* *	*	* *	5/9	55.6%
Rajan P (2013)	*		* *	3/9	33.3%

Rao Deepika PC (2013)	* *	*	*	* *	6/9	66.7%
Zizzi A (2013)	* *		*	* *	5/9	55.6%
Saxena RM (2012)	* *	*	*	* *	6/9	66.7%
Pan Z (2010)	* *	*	*		4/9	44.4%
Wolff RE (2009)	* *	*	* *	* *	7/9	77.8%
Akalin FA (2008)	*	*	*	* *	5/9	55.6%
