# Supplementary figures and images for: Association between Periodontitis and HbA1c Levels in Non-Diabetic Patients: A Systematic Review and Meta-Analysis
Source: Healthcare (Basel). 2023 Sep 28;11(19):2649. doi: 10.3390/healthcare11192649 (PMC10572398; doi:10.3390/healthcare11192649)

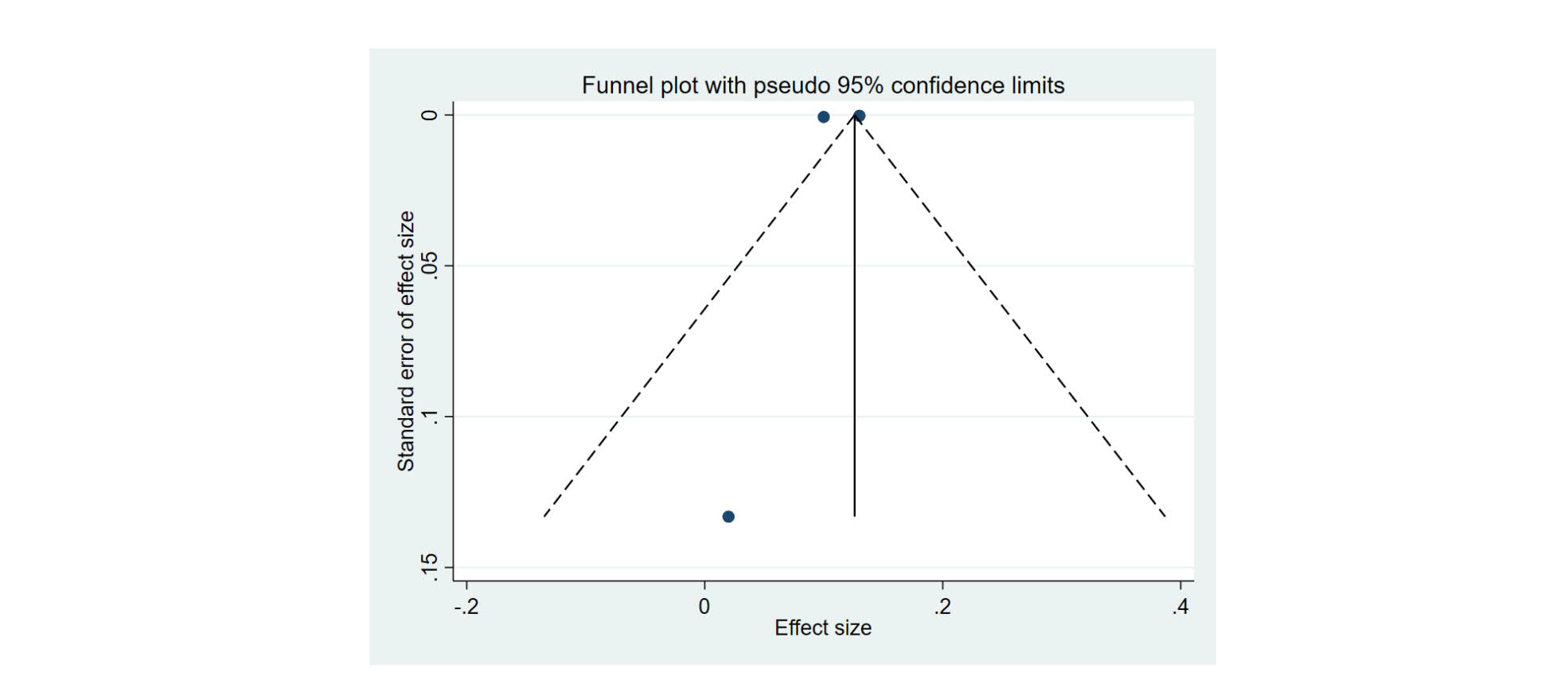

Supplement: Supplementary file 1 [file healthcare-11-02649-s001.zip › Figure S1.jpg]

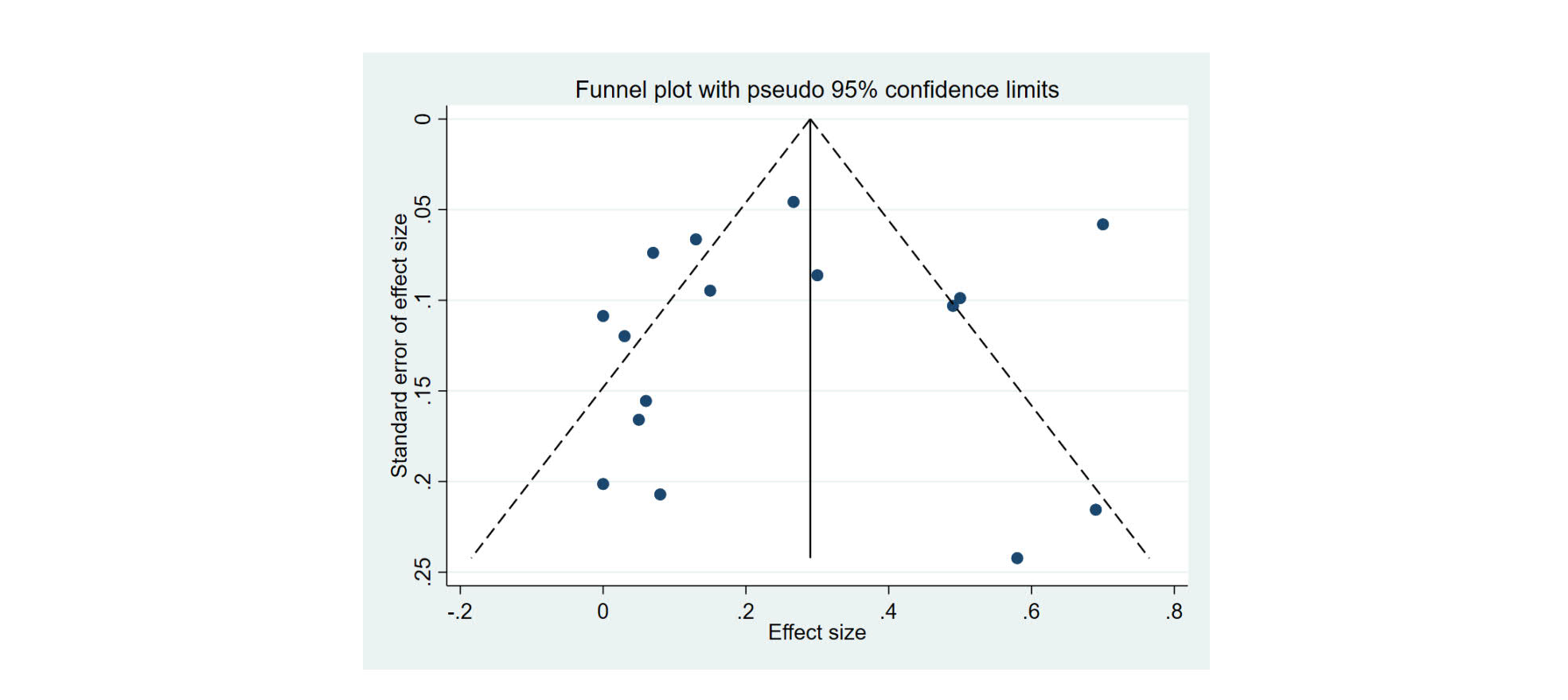

Supplement: Supplementary file 1 [file healthcare-11-02649-s001.zip › Figure S2.jpg]

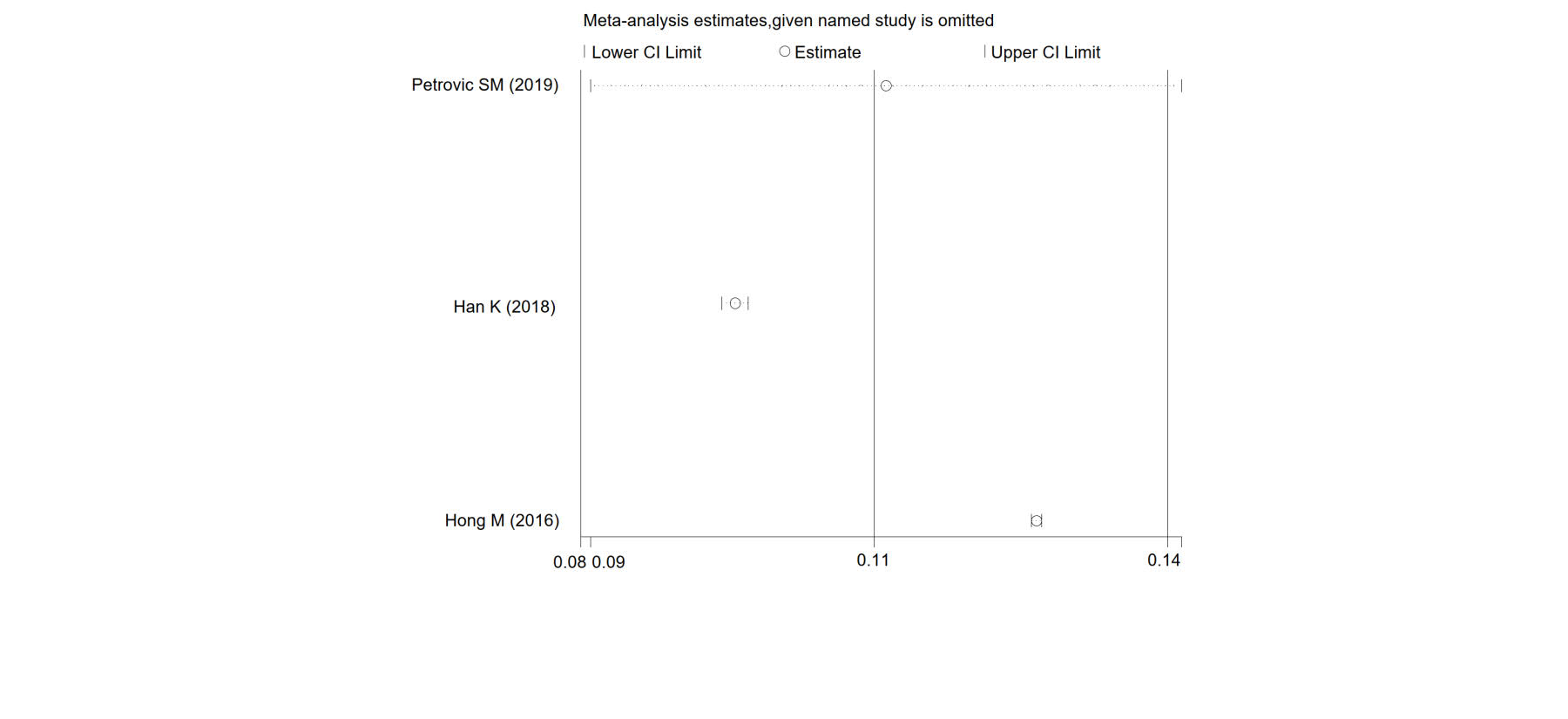

Supplement: Supplementary file 1 [file healthcare-11-02649-s001.zip › Figure S3.jpg]

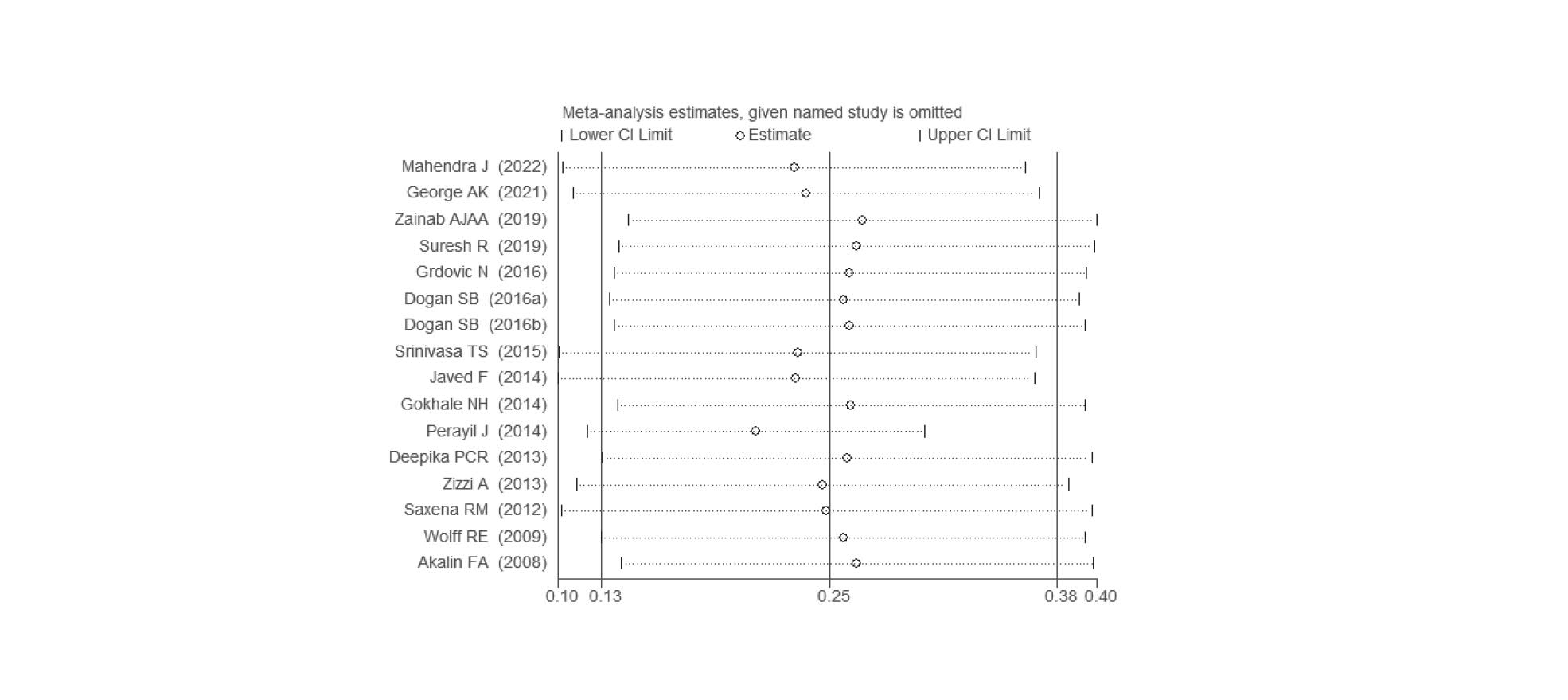

Supplement: Supplementary file 1 [file healthcare-11-02649-s001.zip › Figure S4.jpg]
